# Commercial *Saccharomyces cerevisiae* Yeast Strains Significantly Impact Shiraz Tannin and Polysaccharide Composition with Implications for Wine Colour and Astringency

**DOI:** 10.3390/biom9090466

**Published:** 2019-09-09

**Authors:** Keren A. Bindon, Stella Kassara, Mark Solomon, Caroline Bartel, Paul A. Smith, Alice Barker, Chris Curtin

**Affiliations:** 1 The Australian Wine Research Institute, Hartley Grove, Urrbrae, Adelaide, SA 5064, Australia (S.K.) (M.S.) (C.B.) (P.A.S.) (A.B.) (C.C.); 2 Wine Australia, Industry House, Corner Hackney and Botanic Roads, Adelaide, SA 5000, Australia; 3 Department of Food Science and Technology, Oregon State University, 232B Wiegand Hall, Corvallis, OR 97331, USA

**Keywords:** proanthocyanidin, anthocyanin, pectolytic, pectin, mannoprotein

## Abstract

To gain knowledge on the role of *Saccharomyces cerevisiae* yeast strains (and their hybrids) on wine sensory properties, 10 commercially available yeast strains were selected on the basis of their widespread usage and/or novel properties and used to produce Shiraz wines. Significant differences were evident post-alcoholic fermentation and after 24 months of ageing with regards to the number of wine compositional variables, in particular the concentration of tannin and polysaccharide. Strain L2323 is known for its pectinolytic activity and yielded the highest concentration of both yeast- and grape-derived polysaccharides. Wines made with the mannoprotein-producing strain Uvaferm HPS (high levels of polysaccharides) did not have elevated concentrations of yeast-derived polysaccharides, despite this observation being made for corresponding model fermentations, suggesting that mannoprotein production or retention might be limited by the wine matrix. Wine tannin concentration showed a high level of variability between strains, with L2323 having the highest, and AWRI1503 the lowest concentration. Sensory analysis of the wines after 24 months ageing revealed significant differences between the yeast strains, but only the attributes *opacity* (visual colour) and *astringency* could be predicted by partial least squares regression using the wine compositional data. Notably, the *astringency* attribute was associated with higher concentrations of both tannin and polysaccharide, contrary to reports in the literature which suggested that polysaccharide exerts a moderating effect on astringency. The results confirm previous reports demonstrating that the choice of yeast strain represents an opportunity to shape wine style outcomes.

## 1. Introduction

It is common practice for winemakers to choose specific *Saccharomyces cerevisiae* wine yeasts to achieve desired wine styles, as evidenced by the growing number of strains commercially available for use across the global wine industry. While the domestication signature within wine yeast genomes is not as strong as that evident amongst brewing yeasts, commercial wine yeasts nevertheless display enhanced ‘wine stress resistance’ [1]. Key traits described as important for wine yeasts include low production of sulfides and volatile acidity, an optimal flavour profile, the effective use of nitrogen, temperature and ethanol tolerance [2,3,4,5]. When compared to wild *Saccharomyces*, wine strains are distinct in terms of the flavour and aroma attributes they impart [2]. The importance of flavour outcomes are most commonly cited in the context of white wine production, from both a sensory and consumer perception perspective [3,6,7].

The perception of ‘quality’ or ‘style’ in red wines is significantly more complex, and it is notable that multiple styles can exist which may have equal success in terms of consumer outcomes [8,9]. This may partly account for a lack of literature describing outcomes of fermenting with different commercial *S. cerevisiae* strains in red winemaking, with a limited number of studies giving way to a greater emphasis on the potential of non-*Saccharomyces* yeasts to shape style [10,11,12,13,14,15,16]. As a result, knowledge of red wine sensory and ‘style’ outcomes as a function of the commercial *S. cerevisiae* strain employed presents a gap in the current literature.

In recent years, a key finding of red winemaking studies where yeast strain was a variable is the significant impacts on non-volatiles such as tannin, colour (including anthocyanin) and polysaccharides [14,17,18,19,20,21]. Since both colour and tannin are known properties positively associated with red wine quality [22,23,24], yeast strain selection for optimal extraction and retention of phenolics is a critical step in the winemaking value chain, in particular when considered together with the intrinsic role of the grape source.

Yeast cells have been observed to have the capacity to adsorb and hence remove both wine anthocyanin and tannin [25,26,27,28,29], and this is an important consideration when evaluating the suitability of yeast strains for red winemaking. Furthermore, fermentation-derived or added mannoproteins can precipitate and remove tannin and pigmented material [30,31,32]. In light of this, certain *S. cerevisiae* strains have been identified as mannoprotein ‘overproducers’ [33,34]. While mannoproteins are considered to impart beneficial characteristics in terms of mouthfeel and colour retention [28] the results in wine need to be considered in light of the aforementioned studies, which showed losses of non-volatiles through complexing and concomitant precipitation.

Another way in which yeast can influence the polysaccharide profile of a wine is via expression of pectinolytic activity, in particular endo-polygalacturonases [35]. Yeast-derived polygalacturonases undergo complex regulation in response to the available carbon source as well as genetic (transcriptional, epigenetic) factors, and hence, are variably expressed [35,36,37]. While evidence exists that certain wine characteristics such as turbidity, filterability or juice yield may improve with the use of pectinolytic yeasts [20,35,38] only one study has shown quantitative changes in wine colour and phenolic index using a pectinolytic non-*Saccharomyces* yeast strain [20]. Hence, quantitative information on the impacts of polygalacturonase activity in *S. cerevisiae* strains on wine macromolecules (colour, tannin, polysaccharide) is lacking.

The objective of the presented study was to provide a holistic dataset comparing 10 commercially available *S. cerevisiae* strains in red Shiraz winemaking in terms of fermentation performance, wine volatile and non-volatile profile, and sensory outcomes. Detailed information describing the impact of yeast strain upon wine tannin and polysaccharide composition is presented for the first time. To attempt to understand the mechanisms underpinning some of the observed effects on wine macromolecules, model fermentations were also performed and are discussed. Overall, this work demonstrates the strong impact that selection of commercial *S. cerevisiae* strain alone may have on wine style outcomes, in particular for the sensory attributes *astringency* and *opacity* (colour).

## 2. Materials and Methods

### 2.1. Instrumentation

An Agilent model 1100 HPLC (Agilent Technologies Australia Pty Ltd., Melbourne, Australia) was used with Chemstation software for high-performance liquid chromatographic analyses. For the analysis of wine volatiles an Agilent 6890/7980A gas chromatograph (GC) (Forest Hill, VIC, Australia) equipped with a Gerstel multipurpose sampler (MPS 2XL, Lasersan Australasia, Robina, QLD, Australia) was used, coupled to either an Agilent 355 sulfur chemiluminescence detector (SCD) or an Agilent 5975C VL mass selective detector. GC instrument control and data analysis were performed with Agilent GC ChemStation software and Maestro software integrated version 1.3.3.51/3.3 (Maestro, Schrödinger, LLC, New York, NY, USA).

### 2.2. Yeast Strains

Ten commercial wine strains (Table 1) were obtained and rehydrated according to the manufacturer’s instructions.

### 2.3. Grape Samples and Winemaking Treatments

*Vitis vinifera* L. cv. Shiraz fruit was obtained from the McLaren Vale region of South Australia, in the 2014 season, at a ripeness level of 15 °Baumé. Prior to winemaking, a ≈2 kg sub-sample of grape bunches was obtained for analysis and stored at 4 °C for no longer than 24 h. Fresh grapes were destemmed to obtain a homogenous grape sample and triplicate 200 berry and 50 g samples were collected. The 200 berry samples were frozen at −80 °C for later analysis and the 50 g samples were processed fresh as described below. Thereafter, grapes (50 kg per ferment) were crushed and de-stemmed with the addition of 40 mg/L K_2_S_2_O_5_. Samples of must were analysed for pH, titratable acidity and total soluble solids (as °Baumé) as described below and based on these measurements, a standard addition of tartaric acid was made to each ferment to adjust the pH to 3.6. The must was supplemented with (NH_4_)_2_HPO_4_ to a total assimilable nitrogen content of 200 mg/L. Rehydrated yeasts were inoculated at a concentration of 200 mg/L, in triplicate. Ferments were conducted in a room maintained at 15 °C and the cap was plunged twice a day, with soluble solids and temperature monitored daily. Ferments were drained and pressed when the °Baumé reached 2°. From a sub-set of the wines, a 1 kg sample of pressed marc was collected and frozen at −20 °C until analysed. The free run juice and pressings were further fermented to dryness (≤1 g/L residual sugar) in a room maintained at 20 °C when the wine was then racked off gross lees. At this stage, wine samples were collected for analysis of wine tannin and polysaccharide, as described below. Furthermore, a 200-mL sample of lees was collected from a sub-sample of the wines, centrifuged to remove excess wine, and frozen at −20 °C until analysed. Malolactic fermentation of dry wines was completed at 20 °C following addition of VP41 bacteria (Lallemand, Australia). Tartaric acid was added where required to adjust all wines to a total titratable acidity of 6 g/L, corresponding to pHs within the range 3.5–3.7. K_2_S_2_O_5_ was added at 40 mg/L to prevent spoilage and malolactic fermentation and the wine was cold-stabilised at 0 °C for 21 days. The wine was then racked off fining lees and K_2_S_2_O_5_ was added to ensure a free SO_2_ level of 30 mg/L and total of ≈ 60 mg/L. Wines were filtered through an Ekwip Z6 (0.8 µm) membrane (Winequip, Dudley Park, S.A., Australia) and bottled in 750 mL bottles using Saran-tin screw-cap closures. Wines were stored at 15 °C and full chemical and sensory analyses of wines was performed 24 months after bottling.

### 2.4. Model Fermentations with Added Tannin and Anthocyanin

Shiraz grapes (2014 season) from the Coonawarra region of South Australia were crushed, de-stemmed, and pressed. The juice was retained and stored at −20°C until required. Juice was thawed, then the final sugar concentration adjusted to 220 g/L with D-glucose and D-fructose and the pH was adjusted to 3.32 using a solution of 20% (*w*/*v*) aqueous tartaric acid. Grapex skin tannin solution (GSkinEx, Tarac Technologies, Nuriootpa, South Australia) was added at a rate of 7.5% (*v*/*v*). This addition yielded juice concentrations of 550 mg/L monomeric anthocyanin and 1.2 g/L of tannin, with corresponding measures of 11.2 for wine colour density and 1.04 for non-bleachable pigments, measured as outlined below. The adjusted juice was sterilised by the addition of dimethyl decarbonate to a final concentration of 160 μL/L. Five yeast strains from the winemaking trial (2323, 1503, BDX, EC1118, HPS) were obtained from the Australian Wine Research Institute (AWRI) Wine Microorganism Culture Collection (WMCC). Cryogenically preserved (−80 °C) strains were cultured and maintained on YPD plates (10 g/L yeast extract, 20 g/L peptone, 20 g/L glucose, 16 g/L agar) and stored at 4 °C. Each yeast strain was inoculated into 5 mL YPD medium (10 g/L yeast extract, 20 g/L peptone, 20 g/L glucose) and incubated overnight at 28 °C with agitation. These starter cultures were then transferred (1%, *v*/*v*) into the Shiraz juice (diluted 1:1 with water) and incubated for two days at 22 °C with agitation. A 90-mL aliquot of adjusted Shiraz juice was then inoculated to a starting cell count of 1 × 10^6^ cfu/mL and incubated at 22 °C with stirring (250 rpm). Fermentation progress was monitored by following weight loss. Once fermentation was complete, the wines were cold settled at 4 °C for a minimum of 7 days and sampled for further analysis.

### 2.5. Basic Must and Wine Compositional Analysis

Must samples were obtained immediately after crushing and then centrifuged. Total soluble solids were determined using an electronic refractometer. Must and wine pH was determined using a pH meter and combination electrode. Must and wine TA and was determined by titrating with 0.33 M sodium hydroxide solution to a pH end-point of 7 and 8.2 and expressed in g/L of tartaric acid equivalents. The concentrations of wine ethanol, residual sugar (as the sum of glucose and fructose), glycerol, malic acid, succinic acid and acetic acid were measured by high-performance liquid chromatography (HPLC) using a Bio-Rad HPX-87H column (Biorad, Hercules, CA, USA) as described previously [39]. Briefly, the running isocratic mobile phase was 5 mM H_2_SO_4_ at 0.5 mL/min with the column maintained at 65 °C. Samples were diluted in mobile phase, and 10 µL was injected to the column and monitored by refractive index detection with a total run time of 35 min. Calibration curves relating concentration to refractive index measurements were fitted by least squares regression using Agilent Chemstation software (Agilent Technologies Australia Pty Ltd., Melbourne, Australia).

### 2.6. Marc and Lees Extraction

Frozen marc samples were separated into skin and seed components while kept on ice. Frozen lees samples were defrosted in a water bath at 25 °C for 30 min and mixed with a metal spatula prior to analysis. The fresh weight to dry weight ratio of skin and lees samples was recorded following lyophilization in pre-tared centrifuge tubes. Skins (2 g) and lees (200 mg) underwent two sequential extractions in 70% (*v*/*v*) acetone over a 48-h period, with constant shaking. Prior to extraction, skin samples in solvent were homogenised using an Ultra-Turrax T25 high-speed homogeniser with a S25N dispersing head (Janke & Kunkel GmbH & Co., Staufen, Germany) at 24,000 rpm for 2 min. After extraction, samples were centrifuged and the supernatants were retained and pooled. An aliquot was dried under a stream of nitrogen and reconstituted in 15% (*v*/*v*) ethanol for later analysis of tannin. The acetone-extracted pellets (cell wall material) were retained and dried under vacuum at −50 °C. Dry cell wall material was ground to a fine powder with a mortar and pestle and passed through a 1-mm^2^ mesh, after which 10 mg was weighed into a screw-cap centrifuge tube. A 100-μL aliquot of 12 M sulfuric acid was added to the material and gently mixed, then left to stand at room temperature for 1 h. A 1-mL aliquot of ultrapure water was then added, the sample vortexed, and then maintained at 100 °C in a dry bath for 3 h with intermittent vortexing. Samples were neutralised with NaOH, then diluted as required with MilliQ water prior to monosaccharide analysis as described below.

### 2.7. Tannin Concentration and Colour Analysis

Tannin in extracts and wines, as well as wine colour properties were determined according to a published high-throughput method [40]. For the quantification of tannin in wine, a 25 µL aliquot of grape, marc or lees extract (in 15% (*v*/*v*) ethanol) or wine was mixed with 300 µL of 0.04% (*w*/*v*) methyl cellulose (Sigma Aldrich, St. Louis, MO, USA) solution (treatment) or water (control) in a 96-well plate (1 mL volume), shaken thoroughly, and left to stand for 3 min. Saturated ammonium sulfate (Sigma Aldrich, St. Louis, MO, USA) was then added (200 µL), followed by water (475 µL). Plates were shaken well and left to stand for 10 min, then centrifuged on a Hettich Universal 32 R centrifuge equipped with a Hettich 1645 rotor for 96 well plates (Adelab Scientific, Adelaide, S.A. Australia). The 280-nm absorbance of a 300 µL aliquot of both the treatment and control samples was then measured using a SpectraMax M2 Microplate Reader (Molecular Devices, San Jose, CA, USA). The difference in the 280-nm absorbance between the control and treatment samples was used for tannin quantification using a quantitative standard of (−)-epicatechin (Sigma Aldrich, St. Louis, MO, USA). Standardization of the tannin analysis across the staggered analysis time points was monitored by using a purified commercial seed extract (Tarac Technologies, Nuriootpa, Australia) in each 96-well plate assayed.

### 2.8. Tannin Composition

Solid phase extraction on Oasis HLB (3 mL, 60 mg, 30 μm) cartidges (Waters, Rydalmere, NSW, Australia) was used to purify wine tannin for compositional analysis according to a modification of a published approach [24,41]. Tannin isolates made up in methanol and analyzed by phloroglucinolysis [42] with the modifications described previously [43]. Tannin size distribution was also analyzed by gel permeation chromatography (GPC) using an approach adapted from the original method [44] to allow greater resolution of high molecular mass material, as described previously [45]. Briefly, tannin samples in methanol were diluted with 4 volumes of mobile phase (*N,N*-dimethylformamide containing 1% (*v*/*v*) glacial acetic acid, 5% (*v*/*v*) water and 0.15 M lithium chloride) and 20 µL was injected to two PLgel columns in series: 500 Å followed by 10^4^ Å (both 300 × 7.5 mm, 5 µm) (Varian Inc., Mulgrave, Victoria, Australia). The method was the isocratic method, using the mobile phase described above at a flow rate of 1 mL/min at 60 °C. Tannin elution was monitored at 280 nm. The standards for calibration were according to the published method [43] using a second order polynomial fitted with the cumulative mass distribution at 50% elution for each standard.

### 2.9. Polysaccharide Preparation and Hydrolysis

A 1-mL aliquot of wine combined with 5 mL of absolute ethanol and held at 4 °C for 18 h to facilitate polysaccharide precipitation, then centrifuged at 8000× *g* for 5 min. Thereafter, the supernatant was removed and the pellet retained. Pellets were briefly air-dried to remove excess ethanol, then reconstituted in 800 µL of Milli-Q water. Extracts were then dialysed against 3 changes of Milli-Q water (at 4 °C) using a 1 mL Pur-a-Lyzer dialysis tube of molecular weight cut-off 3500 Da (Sigma-Aldrich, St. Louis, MO, USA). Thereafter, samples were frozen at –80 °C and freeze dried. Dry samples were reconstituted in either 0.1 M sodium nitrate or 2 M TFA. Samples prepared in 0.1 M sodium nitrate were analysed by size exclusion chromatography according to a published method [46]. Samples in 2 M TFA were hydrolyzed at 100 °C for 3 h, cooled on ice, concentrated under vacuum at 30 °C (Heto vacuum centrifuge, Heto-Holten A/S, Allerod, Denmark). Samples were made up in Milli-Q water for monosaccharide analysis, described below.

### 2.10. Monosaccharide Analysis

Free monosaccharides in wine (diluted 1:10 with water), as well as those released in polysaccharide and cell wall hydrolysates were quantified using an adaptation of a published method [47]. Internal standards used were deoxy-glucose for wines (which contain free ribose) and ribose for polysaccharide hydrolysates (Sigma-Aldrich, St. Louis, MO, USA). A 25-µL of sample containing internal standard was added to 96.2 µL of derivatising reagent (0.5 M of methanolic 1-phenyl-3-methyl-5-pyrazolone (Sigma-Aldrich, St. Louis, MO, USA) in 1 M NH_4_OH). The sample was vortexed heated at 70 °C for 1 h in a heating block. Thereafter, samples were cooled on ice and neutralised with formic acid. Samples were then extracted twice with dibutyl ether (Sigma-Aldrich, St. Louis, MO, USA) and the upper layer was discarded, and residual dibutyl ether was then removed under vacuum at room temperature. Derivatised monosaccharides were quantified by HPLC using a C18 column (Kinetex, 2.6 μm, 100 Å, 100 × 3.0 mm) fitted with a guard cartridge (KrudKatcher Ultra HPLC in-line filter, 0.5 μm) (Phenomenex, Lane Cove, NSW, Australia). The mobile phases were solvent A, 10% (*v*/*v*) acetonitrile in 40 mM aqueous ammonium acetate, and solvent B, 70% (*v*/*v*) acetonitrile in water. The following linear gradient was used: for solvent A (with solvent B making up the remainder) 92% at 0 min, 84% at 12 min, to 0% at 12.5 min, 0% at 14 min, then returning to the starting conditions at 14.5–18.5 min, 92%. A flow rate of 0.6 mL/min was used with a column temperature of 30 °C. The PMP-monosaccharide derivatives were monitored and quantified at 250 nm. Monosaccharides were and identified and quantified using commercial standards (Sigma-Aldrich, St. Louis, MO, USA).

### 2.11. Wine Volatiles

Fermentation products in wine samples were analysed by gas chromatography-mass spectrometry (GC-MS) according to a published method [48]. Briefly, wine was diluted 1:10 with buffer (5 g/L potassium hydrogen tartrate, pH 3.2 with tartaric acid), with NaCl added to 20% (*w*/*v*). Labelled internal standard was added as described previously [48]. Samples were heated to 35 °C for 5 min with agitation, and a solid-phase-micro-extraction fibre (polyacrylate) was introduced to the sample headspace for 10 min. Adsorbed volatiles were then desorbed within the injector (splitless mode) for 10 min at 260 °C. The GC was fitted with a 60 m × 0.25 mm wax column of 0.25-µm film thickness. Helium (ultra-high purity) was used as the carrier gas in constant flow mode. The oven temperature was started at 35 °C, held at this temperature for 2 min then increased to 150 °C at 5 °C/min, and held at this temperature for 2 min, then finally increased to 230 °C and held for 5 min. The total run time was 42 min. The MS quadrupole temperature was set at 150 °C, the source was set at 230 °C and the transfer line was held at 260 °C. Positive ion electron impact spectra at 70eV were recorded in selective ion monitoring (SIM) and scanning (SCAN) modes with a solvent delay of 5 min.

Low molecular weight sulfur-containing volatiles in wine samples were analysed according to another published approach [49]. Briefly, internal standards ethylmethyl sulfide and propyl thioacetate were added to wine samples containing NaCl at 20% (*w*/*v*) to a final concentration of 50 μg/L under temperature control (4 °C). Sample vials were heated from 4 to 45 °C for 30 min with continuous stirring. Headspace sampling was conducted using a Gerstel 1.0 mL HS syringe (Lasersan) fitted with a custom-made dual gauge cone-tip needle (0.47 mm/0.63mm, SGE, Ringwood, VIC, Australia), and the syringe heating block was held at 60 °C. A 100-μL static headspace sample was injected into the cool-on-column inlet COC inlet at 10 μL/s. The syringe was purged to atmosphere with nitrogen at 10.34 kPa (BOC grade 3.5) for 3 min after injection. GC-SCD was performed using a 15 m × 0.25 mm FactorFour VFWAXms fused silica capillary column, 0.50 μm film thickness (Varian, Mulgrave, VIC, Australia) connected with a fused silica universal straight connector (Grace Davison Discovery Sciences) to a 60 m × 0.25 mm VICI ValcoBond VB-5 fused silica capillary column, 0.50 μm film thickness (Chromalytic Technology, Boronia, VIC, Australia), with a 2 m × 0.53 mm retention gap. Helium (ultra-high purity), linear velocity = 37 cm/s, flowrate = 2.7 mL/min in constant flow mode, was used as the carrier gas. The initial oven temperature was held at 5 °C for 5 min, increased to 150 at 5 °C/min, and held at this temperature for 5 min. The cool-on-column inlet (Agilent G3440A) (pressurised to 252.69 kPa) was held at 30 °C for 10 min and ramped at the same rate as the oven. The oven and inlet were cryogenically cooled with liquid nitrogen. SCD detection was carried out using sulfur trap gas purifiers on all gas lines, with the detector base temperature held at 200 °C and the dual plasma controller at 800 °C. The reagent gases were air (instrument grade), 60.0 sccm; hydrogen (ultra-high purity), 45.0 sccm; and ozone, generated in situ from air at 41.37 kPa.

### 2.12. Quantitative Descriptive Sensory Analysis

Two of the three fermentation replicates for each treatment were chosen for sensory analysis based on either their sensory attributes from the informal preliminary assessment or basic chemical data. A panel of nine assessors (three male, six female) with an average age of 52 years (standard deviation, SD = 12.7) was convened for this study, all of whom were part of the AWRI trained descriptive analysis panel. Assessors attended one training session to familiarise themselves with the descriptors and to determine whether the list of descriptors needed to be adjusted. Wines were assessed by appearance, aroma and palate. Standards for aroma attributes were presented and discussed and these standards were also available during the booth practice session and the formal assessment sessions. Following the training session, tasters participated in a practice session in the sensory booths under the same conditions as those for the formal sessions. After the practice session, any terms which needed adjustment were discussed and the final list of terms determined. For the formal session, this list was refined to include one appearance term, twelve aroma terms (eleven defined and “other”), and fourteen palate terms (thirteen defined and “other”). These attributes, definitions/synonyms and standards are provided in the Supplementary Information (Table S1). Samples were presented to panelists in 30-mL aliquots in 3-digit-coded, covered, ISO standard wine glasses at 22–24 °C, in isolated booths under daylight-type lighting, with a randomised presentation order, except in the practice sessions, where there was a constant presentation order. All samples were expectorated. The assessors were forced to have a 30 s rest between samples and a 10-min rest between trays. During the 10-min break, assessors were requested to leave the booths. Twenty wines were evaluated during this study, presented to assessors three times in a modified Williams Latin Square incomplete random block design generated by Fizz sensory acquisition software (version 2.47B, Biosystemes, Couternon, France). The twenty wines were split into five blocks of four wines. Panelists assessed four blocks per session. Formal assessment took place over four sessions. The intensity of each attribute was rated using an unstructured 15-cm line scale from 0 to 10, with indented anchor points of ‘low’ and ‘high’ placed at 10% and 90% respectively. Data was acquired using Fizz sensory software. Panel performance was assessed using Fizz, Senstools (OP & P, Utrecht, The Netherlands) and PanelCheck (Nofima, Tromsø, Norway) software, and included analysis of variance for the effect of judge and presentation replicate and their interactions, degree of agreement with the panel mean and degree of discrimination across samples. All judges were found to be performing to an acceptable standard.

### 2.13. Statistical Analysis

Analysis of variance (ANOVA) and post-hoc significance tests were carried out using either the Minitab (Minitab Inc., Sydney, NSW) or JMP 5.0.1 (SAS, Cary, NC, USA) statistical software packages. For sensory analysis, the effects of yeast (Y), judge, fermentation replicate nested in yeast (FRep(Y)), yeast and fermentation replicate nested in tasting replicate (TRep(Y,FRep)), the interaction between judge and yeast (J*Y), and the interaction between judge and fermentation replicate nested in yeast were assessed (J*FRep(Y)). Principal component analysis (PCA) and partial least squares regression (PLS) were performed using the Unscrambler X10.3 (CAMO Software, Oslo, Norway). Data was scaled as the inverse of the standard deviation for multivariate analyses. All PCA and PLS analyses were performed with cross validation.

## 3. Results and Discussion

### 3.1. Fermentation and Basic Wine Composition

The progress of sugar consumption during fermentation (expressed in g/L of sugar) with associated fermenter temperatures is presented as Supplementary Information (Figure S1). Differences in the rate of fermentation were observed between strains estimated by the days to drop below 36 g/L sugar (<2 °Baume), which was significant by one-way ANOVA (*p* < 0.001, not shown). Strains HPS and NT50 reached the late fermentation stage most rapidly, within 6 days, significantly faster than strains RX60 and 1503 which took 10 days. The remaining yeast strains were intermediate in fermentation rate and were not significantly different from one another, as determined by a post-hoc Student’s *t*-test. Temperatures within fermenters changed over time due to the exothermic nature of alcoholic fermentation, peaking at day 5 for all strains, except 1503, which peaked at day 6. Following pressing (at 36 g/L sugar), temperatures were maintained at 22 °C, with no significant differences between stains observed during this period.

The basic compositions of wines made with the 10 strains differed significantly for all parameters but malic acid concentration and titratable acidity (Table 2), albeit within relatively narrow ranges of values. Final residual sugar levels did not necessarily relate to the initial fermentation rate. Wine made with RX60 had similar residual sugar to the other strains, whereas 1503-fermented wine retained significantly higher concentrations of residual sugar (~1 g/L). Differences in final alcohol concentration may represent variation between strains in terms of alcohol yield. Although there were differences in the initial sugar of the Shiraz must across the range of ferments after fruit randomization, the treatment triplicates were not significantly different when compared using one-way ANOVA.

### 3.2. Yeast-Derived Flavour Compound Production

Yeast strain significantly affected the production of esters, higher alcohols, volatile fatty acids (Table S2) and volatile sulfur compounds (Table S3). A principal component analysis of these data (Figure S2) showed that the greatest separation of strains was along PC1, which explained 44% of the variation, was driven by differences in the abundance of ethyl esters and corresponding medium chain volatile fatty acids (strains 796 and RX60) versus branched chain fatty acids, their corresponding ethyl esters and higher alcohols, and volatile sulfur compounds (BDX). Separation along PC2, which explained a further 24% of variation, was largely driven by relative concentrations of 2-methylpropyl acetate and ethyl propanoate (highest for strains EC1118, NT50, 1503) compared with 2-methylbutyl acetate, 3-methylbutyl acetate and 2-methyl propanoic acid (HPS, 2323). Yeast strain CLOS yielded wine with an intermediate profile of the aroma compounds analysed. Of the strains clustered near to one another in terms of their flavour phenotypes [3], it was interesting to note that EC1118, NT50 and the *S. cerevisiae* component of hybrid strain 1503 all belong to the ‘Prize de Mousse’ clade according to whole genome sequences [50]. Whole genome comparison revealed high levels of inbreeding and strain redundancy across the spectrum of commercial wine strains of *S. cerevisiae* [50]. Conversely, RX60 and 796 were genetically distinct, as were 2323 and HPS.

### 3.3. Wine Tannin Composition and Colour Properties

The potential impact of yeast strain on red wine phenolics has been well-documented by other researchers, and can affect both tannin concentration and colour properties [17,18,19]. However, the mechanisms by which yeast strain can alter wine phenolic composition are poorly understood, and may involve direct impacts on extraction (ethanol, cell wall breakdown), or differences in the adsorption of tannin or coloured compounds by yeast cells [26,29,51,52,53]. A further possibility is that yeast mannoproteins may remove tannin from wine as an insoluble precipitate [30]. Our study aimed to explore the range of tannin and colour differences achievable from a given grape source simply by *S. cerevisiae* strain selection and also sought to identify potential mechanisms by which the strains exert these effects. Table 3 shows the results for both the concentration and compositional properties of wine tannin and colour produced by the 10 yeast strains studied at 2 years of bottle age, the point which corresponded to the wine sensory analysis. Wine tannin concentration differed markedly between yeast strains and was slightly lower at the 2-year analysis point relative to that measured at the end of fermentation (data not shown), but relative treatment effects were maintained with wine ageing. Increased wine tannin concentration was weakly associated with higher wine colour density (*R*^2^ = 0.61, *p* < 0.01). Strain 2323 produced wines with the highest tannin concentration and wine colour density, corresponding to elevated non-bleachable pigment concentration. Some yeast strains produced wines with similar tannin concentration and colour density to strain 2323, and were CLOS, EC1118 and F15, but these strains were not as well discriminated by the statistical analysis from the other strains studied as was 2323. HPS, RX60 and 1796 had intermediate tannin concentration, but did not necessarily show the higher wine colour densities observed for the strains which had significantly higher tannin concentration. Wines made with yeast strains 1503, BDX and NT50 had the lowest tannin concentrations, although again these were not completely discriminated by the ANOVA from the strains producing intermediate tannin levels. Interestingly, despite having lower tannin, the wine made with strain NT50 had a wine colour density equivalent to the strains which produced the highest colour.

In terms of tannin compositional differences, a small effect of yeast strain on tannin molecular mass measured by phloroglucinolysis was found. A higher tannin molecular mass was associated with a higher degree of trihydroxylated subunits (*R*^2^ = 0.73), potentially indicating that differences in skin tannin extraction or retention were exerted by the respective yeast strains, resulting in a larger average polymer length (mDP). Since the degree of mass conversion, which indicates the extent to which the tannin could be depolymerised during phloroglucinolysis, was low (at 30% or less), tannin molecular mass determined by GPC provides a better indication of tannin size distribution (hydrodynamic volume) than the phloroglycinolysis assay, especially in aged wines. The GPC results showed minor effects on tannin size, with yeast strains producing wines with the lowest tannin concentration, also generally having a lower molecular mass. However, as for the results on both tannin concentration and colour, the ANOVA did not clearly discriminate the strains in terms of molecular mass (both GPC and phloroglucinolysis). Hence, no correlation of molecular mass and tannin concentration was observed.

In order to understand the potential mechanism by which yeast might affect tannin concentration in wine, two experimental approaches were followed to observe whether differences in yeast strain adsorption (hence removal) properties for tannin exist. For the small-scale experimental wines, marc and lees samples were collected from a sub-set of the 10 yeast strains studied, including the yeast strains which resulted in wines with relatively high and low tannin concentrations: 2323 and 1503. Through analysis of the tannin bound per unit dry weight of acetone-extracted marc and lees material (Supplementary Information Figure S3), it was found that the concentration of tannin per unit dry weight of lees was not significantly different between the strains studied. Due to the scale of the fermentations, it was not possible to obtain an accurate recovery of the yeast lees for each of the ferments. For the analysis of marc samples, stronger differences between the yeast strains were observed. It was found that wines with a high tannin concentration also had higher concentrations of residual tannin in the marc, between 55 and 65 mg/g dry weight. Strain 1503, with the lowest wine tannin concentration, had a significantly lower concentration of residual tannin, at 44 mg/g (Supplementary Information Figure S3). A negative relationship between marc tannin concentration and wine tannin concentration would enable the suggestion that yeast strains affected tannin extraction. Conversely, the positive relationship for marc and wine tannin may point to a relative loss of marc cell wall material during the fermentation, resulting in a greater proportion of residual tannin. Two pertinent questions which could be further addressed were raised based on the tannin composition and recovery results: a. the effect of yeast strain on cell wall degradation, and b. yeast lees yield adsorption properties for tannin, and were further studied by compositional analysis of marc and model fermentations with added polyphenols respectively. The results are detailed in subsequent sections.

### 3.4. Wine Polysaccharide and Monosaccharide Composition

The process of fermentation facilitates the extraction of significant quantities of cell-wall derived polysaccharides from grape skins and pulp [54]. Yeast strains are known to possess different levels of enzyme activity to degrade cell wall polysaccharides, notably, the presence of endo-polygalacturonase activity [20,35,37,38,55,56]. Of the strains used in the current study, 2323 was reported to possess endo-polygalacturonase activity, as was EC1118 and, to a lesser extent, NT50 [37]. The expression of polygalacturonase activity under winemaking conditions is limited by the medium, being inhibited by glucose for example, and being promoted by the presence of polygalacturonate or galactose [37]. Although these activities are noted in various yeast strains, the impact on wine properties is poorly understood, having been observed to impact colour either positively [38] or negatively [57] with effects on wine polysaccharide and tannin composition being as yet non-significant, or unknown. Other effects of yeast strain on polysaccharide composition have been observed, for example the HPS strain used in this study is considered to be an overproducer of mannoprotein [33].

The current study sought to investigate the impact of yeast strain on wine polysaccharide composition, but also to observe the cell wall-degrading effects of yeast through a compositional investigation of the marc and lees collected from a subset of the experiments. The results for the polysaccharide and free monosaccharide concentration and composition of wine are shown in Table 4. Strain 2323 had the highest polysaccharide concentration of all the strains studied, and this was associated with higher concentrations of both yeast-derived (mannose, some glucose) and grape-derived polysaccharides. Associated with this increase in polysaccharide was an elevated concentration of free galacturonic acid in the wine, at a relatively high level of ≈1.1 g/L. We have previously noted elevated levels of free galacturonic acid of ≈1.5 g/L in Shiraz wines prepared with a pectolytic (polygalacturonase-rich, likely to be exo- and endo-polygalacturonase) enzyme preparation, in the order of three times that of the control fermentation [32]. In the current study, free galacturonic acid ranged from 0.5 to 1.1 g/L and may reflect the combination of endogenous grape-derived pectolytic enzyme activity in combination with yeast-derived polygalacturonase. Equivalent levels of free galacturonic acid to strain 2323 were also found for yeast strains 1503 and RX60 (Table 4). Considering the high relative concentrations of monomeric galacturonic acid, this may indicate the presence of elevated polygalacturonase activity in these yeast strains relative to others, for example NT50 is known to have a lower activity than 2323 [58]. Strains 1503 and RX60 would need to be further evaluated to confirm pectinase activity.

A surprising result was that the HPS strain had low total polysaccharide and associated mannose, although this strain is expected to produce higher levels of mannoprotein [33]. To confirm the results for HPS mannoproteins relative to the other strains, purified Shiraz wine polysaccharides were also assessed using peak areas obtained following size exclusion chromatography (Supplementary Information Figure S4). Differences in peak area confirmed that Shiraz wines made using HPS indeed had lower polysaccharide concentration than 2323 and EC1118. This highlights that the response of HPS in terms of polysaccharide production is likely to be dependent upon the ferment matrix conditions.

**Figure 1:**
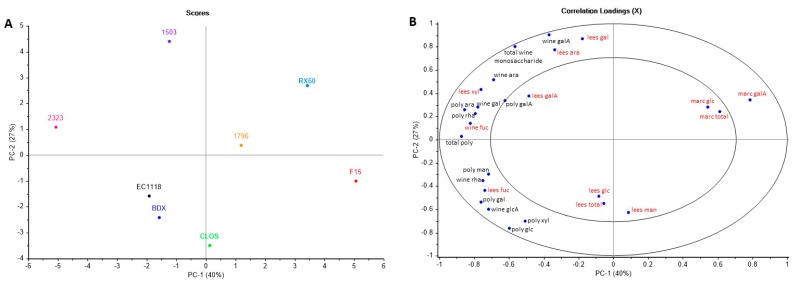
Principal component analysis of the composition of wine soluble (wine) polysaccharide-associated (poly) monosaccharides, and hydrolytically-released monosaccharides from purified lees and marcs of 8 commercial yeast strains (**A**) Scores plot showing each yeast strain as a different colour; (**B**) Correlation loadings plot for wine composition (black) and lees or marc composition (red). Abbreviations: man = mannose, rha = rhamnose, glcA = glucuronic acid, galA = galacturonic acid, glc = glucose, gal = galactose, xyl = xylose, ara = arabinose, fuc = fucose.

To better account for the differences in polysaccharide composition between yeast strains, the monosaccharide composition of lees and marc of a sub-set of 8 strains (excluding NT50 and HPS) was analysed and the results are shown in Table 5. For better visualisation of the results, a PCA of the combined wine, lees and marc data was performed (Figure 1). The greatest variation between yeast strains was defined by PC1, accounting for 40% of the variance in the data. PC1 was defined positively by the concentration of residual galacturonic acid in the grape marc cell walls (Figure 1B), and separated strain F15 from the others (Figure 1A). Strain F15 also had lower total polysaccharide, and polysaccharide-associated galacturonic acid and arabinose which were negatively loaded of PC1. Strongly associated with PC1 was strain 2323, which, as described previously, had higher total polysaccharide and polysaccharide-associated galacturonic acid. It was interesting to note that 2323 also had somewhat lower galacturonic acid in the marc cell walls (Table 5). However, strains BDX, CLOS and EC1118 also had relatively lower galacturonic acid in the marc cell walls but did not necessarily have corresponding differences in wine polysaccharide composition, being negatively associated with PC2, which accounted for a further 27% of the variance in the data. BDX, CLOS and EC1118 also had lower monomeric galacturonic acid in the wine. As highlighted previously, strains 2323, 1503 and RX60 produced higher concentrations of monomeric galacturonic acid and were positively associated with PC2. Since the polysaccharide composition of the lees was found to be primarily mannose and glucose (80–89% by molar proportion) (Table 5), the contribution of grape-derived polysaccharide to the lees was minor. However, some variation in lees composition was also found to be important within the PCA model, accounting for a further 16% (PC3) and 8% (PC4) of the variance (Supplementary Information Figure S5). The grape-derived polysaccharides, which were most abundant in the lees, were rich in arabinose and galactose, accounting for between 7 and 12% of the total by molar proportion which may indicate a loss of extracted PRAGs within the lees at the end of ferment. Strain 1503 had higher lees-associated arabinose, galactose and galacturonic acid (positively loaded on PC2). This potentially indicates that strain 1503 facilitated extraction of polysaccharide material from the grape, as indicated by lower concentrations of these monosaccharides in the marc (Table 5). However, this may also reflect a relatively lower contribution of yeast-derived polysaccharides to the lees. For the additional information explained by PC3 (Supplementary Information Figure S5), this defined strains 2323 and 1796 which had higher concentrations of lees-associated polysaccharide, in particular glucose.

The PCA results show that the respective yeast strains could be clearly discriminated based on the polysaccharide composition of wine, lees and marc. However, this approach did not clearly define the mechanism by which yeast conferred these differences. For some strains, some strong evidence was provided for cell wall degrading activity, for example, strain 2323 both extracted greater quantities of polysaccharide and partially cleaved this material to galacturonic acid. Strain 1503 appeared to have a similar conversion of polysaccharide to galacturonic acid, yet some extracted polysaccharide material was lost as lees. F15, on the other hand, demonstrated a reduced extraction from the grape, without strong evidence that grape-derived polysaccharide was elevated in the lees. These observations were limited by the fact that the % recovery of lees was unknown, hence, differences in yeast biomass production relative to grape-derived lees material could not be deduced. This was further assessed using model fermentations on a sub-set of strains and will be discussed in the next section.

### 3.5. Model Fermentations

To further address questions relating to yeast lees yield and tannin adsorption properties, model fermentations were also performed. This enabled yeast lees formation and tannin/colour adsorption properties of a sub-set of the strains studied to be accurately quantified. Five yeast strains from the winemaking trial (2323, 1503, BDX, EC1118, HPS) were selected for further assessment. From a 1.2 g/L tannin addition to the must at the start of fermentation, the final concentrations of tannin in the finished model fermentations were ≈ 0.9 g/L, and were not significantly different between the strains assessed (Supplementary Information Table S4), indicating that approximately 25% of added tannin was removed by yeast. Effects on wine colour required a more complex interpretation, since yeast metabolites play a role in the formation of non-bleachable (polymeric pigments) but can also adsorb and remove anthocyanin. Relative to the starting addition of colour (control), completed ferments had a 34% loss of anthocyanin, and a 36% loss of wine colour density. Non-bleachable pigments on the other hand, increased by 36%. The net loss of wine colour density presents a likely loss via association with yeast cells, as observed by others [51], but no differences were observed between the strains studied. Based on these observations, the model fermentations did not demonstrate differences in the adsorption of tannin and/or colour which could be used to explain the range of tannin concentration and colour achieved in the experimental Shiraz wines using different yeast strains.

A clear effect of yeast strain on grape-derived polysaccharide component was also not evident in the model fermentations. Since there was no skin contact during the model fermentation, there was no additional source of grape polysaccharide other than the juice itself. The juice polysaccharide pre-ferment was primarily arabinose and galactose in equal proportion (60%), with lower contributions of rhamnose (11%) and galacturonic acid (9%) and this was not affected by fermentation with the various yeast strains. This does not necessarily indicate a lack of polygalacturonase activity in the strains, since the low contribution of galacturonic acid, and higher proportion of rhamnose may be indicative of the presence of rhamnogalacturonan [59] which is resistant to the action of the enzyme.

A striking response of the various yeast strains in the model fermentations was the total polysaccharide recovered in the lees (Supplementary Information Table S4). Strains 1503 and EC1118 had the lowest lees polysaccharide recovery, and 2323 the highest. The trend in polysaccharide lees recovery was associated with differences in glucose, while no differences in lees-associated mannose were found. Although a small amount of cellulose from the grape cell wall material in the juice may have accounted for some lees-associated glucose, this contribution would be expected to be consistent between treatments, and minor given that other grape-derived monosaccharides to lees-associated polysaccharide were low (<15%). Furthermore, the differences in lees polysaccharide composition between strains in the model ferments were also observed in the Shiraz wines (Table 5) where glucose was highest in 2323 lees and lowest in 1503 or EC1118 lees. This result may therefore explain, in part, the separation of yeast strains described following PCA in Figure 1. In the Shiraz wines, lower contributions of yeast-derived polysaccharide to lees in strain 1503 (PC2) may explain the higher proportion of grape-derived polysaccharide associated with the lees. The separation of 2323 from the other strains on PC3 (Supplementary Information Figure S5) was due to a higher contribution of glucose to the yeast lees.

### 3.6. Wine Sensory Properties

**Figure 2:**
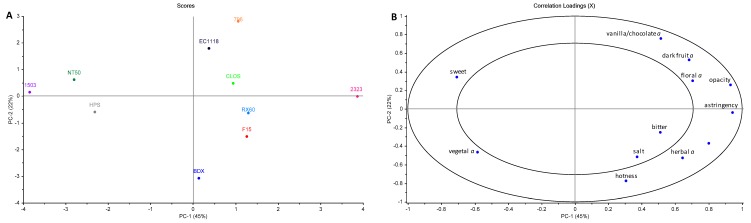
Principal component analysis of the composition of wine sensory attributes determined for duplicate wines prepared using 10 commercial yeast strains (**A**) Scores plot showing each yeast strain as a different colour; (**B**) Correlation loadings plot for wine sensory attributes (aroma variables designated a).

According to the ANOVA, there were ten attributes that differed significantly between yeasts: *opacity, dark fruit aroma, herbal aroma, vegetal aroma, viscosity, sweet taste, salt, astringent, hotness,* and *bitter taste* (Supplementary Information Table S5). Two other attributes were very close to significant (*p* < 0.10, *floral aroma* and *vanilla/chocolate aroma*). PCA was performed using the significant and near-significant sensory attributes (*p* < 0.10) found in the ANOVA. For this dataset, the first two PCs were important in showing the variation in the dataset. Figure 2 shows the PCA scores and loadings for PC1 and 2, which account for a total of 67.2% of the variation in the data set. PC3 accounted for a further 10.9% of the variation in the data set (not shown). Figure 2 shows that yeast strain 2323 was rated higher in *astringency, dark fruit aroma, viscosity* and *opacity*, as seen along PC1, in comparison with 1503, NT50 and HPS rated lower in these characteristics and higher in *sweet taste* and *vegetal aroma*. BDX had the highest ranking for *vegetal aroma.* Strain 1503 had the highest residual sugar (Table 2) and was also found to be rated highest in *sweet taste*. On the other hand, PC2 shows the yeast strains BDX and F15 to be rated higher in *hotness* and *herbal aroma*, whereas 1796 was rated lower in these characteristics and highest in *dark fruit aroma* and *vanilla/chocolate aroma*. BDX, F15 and EC1118 were rated more highly in *bitter taste.* Yeast strains RX60 and CLOS were observed to be rated moderately in most attributes.

### 3.7. Modelling the Yeast-Derived Predictors of Wine Sensory Properties

In order to model the relationship between yeast-associated changes in wine composition and sensory outcomes, PLS was applied. Initially when PLS2 was applied where all significant sensory attributes were included, two attributes—astringency and opacity—were well modelled by the chemistry data. Thereafter, PLS1 was applied to each sensory attribute alone, and the same result as found for PLS2 was observed with the exception that better *R*^2^ values for calibration and validation of 0.57 and 0.46 were respectively obtained for the attribute *vegetal aroma*. This attribute differed significantly between the yeast strain treatments and showed greater variability than other sensory attributes (Supplementary Information Table S5). As discussed previously, BDX was rated more highly in *vegetal aroma* and was associated with an elevated concentration of dimethyl sulfide (Supplementary Information Table S4). *Vegetal aroma* showed the strongest positive relationship with dimethyl sulfide in the PLS1 model and was negatively related to ethyl octanoate, acetic acid and ethyl acetate (results not shown).

**Figure 3:**
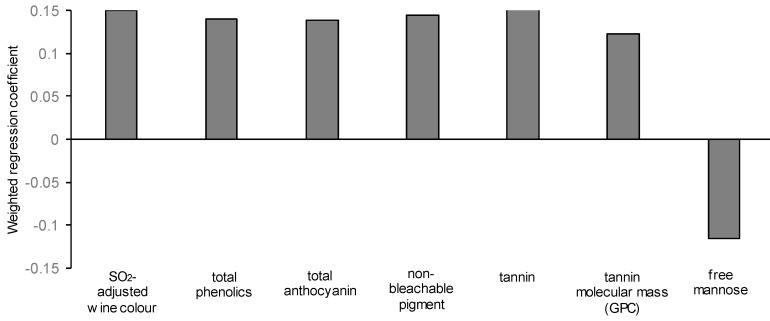
Weighted regression coefficients determined from partial least squares regression (PLS1) analysis of significant wine compositional variables to predict the sensory attribute opacity.

To describe the yeast-associated contributors to the sensory attributes *opacity* and *astringency*, PLS1 models were developed for these attributes and are shown in Table 6. The models were improved using a sub-set of significant variables, and for both the *opacity* and *astringency* attributes, these included wine pH and TA. Although TA was adjusted to ≈6 g/L for all wines and was not statistically different between yeast strains (Table 2), there were small but significant differences in wine pH. Due to the importance of pH in the expression of wine colour and astringency, it was necessary to determine whether these pH differences were critical to the PLS models developed. More PLS models which excluded pH and TA were developed (Table 6) and the results were unchanged, indicating that the minor differences in pH, while significant, were unlikely to be contributing strongly to the perceived *opacity* or *astringency* of the wines. For the prediction of *opacity* from wine compositional data, a model could be developed based on only a few factors, for which the weighted regression coefficients are shown in Figure 3. Since *opacity* describes the optical density of the sample, it is likely to be related to nonvolatile compounds which absorb strongly in the visible light range. Therefore, as expected, opacity was positively correlated with wine colour density, phenolics, anthocyanin, non-bleachable pigment and tannin concentration. Tannin molecular mass (by GPC) was also a significant predictor of opacity. However, as discussed previously, the effects of yeast strain on tannin size were minor.

**Figure 4:**
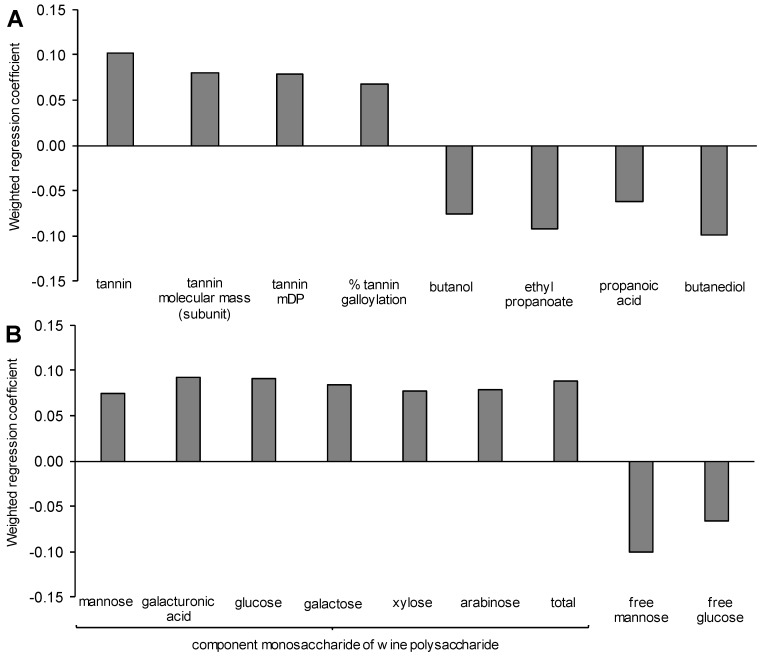
Weighted regression coefficients determined from partial least squares regression (PLS1) analysis of significant wine compositional variables to predict the sensory attribute astringency showing (**A**) tannin composition and fermentation products; (**B**) free and polysaccharide-associated monosaccharides.

For the attribute *astringency*, it was found that certain yeast fermentation products were negatively associated in the PLS1 model (Figure 4A), although there was no evidence from the literature that these should have a textural impact at wine-like concentration and more likely are discriminating metabolites between yeast strains. However, in strong agreement with other studies [60,61,62], tannin measures were found to have a positive association with *astringency* in the PLS1 model developed, with tannin concentration having the greatest contribution, followed by tannin mDP and molecular weight (phloroglucinolysis), as well as the % tannin galloylation (Figure 4A). The linear relationship of tannin concentration and astringency of the averaged replicates gave an *R*^2^ value of 0.7, although there were clear outliers to the curve for BDX and CLOS (results not shown) with these strains having a somewhat higher astringency, but low and high tannin concentrations, respectively. If these strains were excluded from the linear regression, the *R*^2^ for the relationship between tannin concentration and *astringency* was 0.94 (results not shown). Investigating the model further (Figure 4B), an unexpected finding was that all the wine polysaccharide classes were positively associated with *astringency*. It could be seen that BDX, with lower tannin, had relatively high polysaccharide, in particular that containing mannose (Table 3; Table 4) which predicted, in part, its higher relative astringency. CLOS, on the other hand, had lower overall polysaccharide, mainly a reduced contribution of mannose-based material, which appeared to contribute to a lower overall astringency despite its high tannin concentration. This contradicted the reports in the literature which proposed that tannin-mannoprotein interactions may contribute to reduced astringency by reducing the available binding sites for interaction with salivary proteins [28,63]. Although mannoprotein-tannin effects on astringency have been inferred to exist in wine by reference methods, e.g., the gelatin index [28,63], only one study has claimed supplementary evidence for this hypothesis, demonstrating a negative correlation between wine polysaccharides and astringency in Tempranillo wines [63]. It is noteworthy that the published study used commercial wines of different ages, prepared under different conditions, but which also had a far wider range of perceived astringency, tannin and polysaccharide concentration when compared with the narrow range we present here made from a single fruit source. However, in the study by [63], the finalised multivariate model for astringency prediction did not include mannoprotein or rhamnogalacturonan II, suggesting this was due to a non-linear relationship with astringency. Previous research by our group has previously shown a similar positive correlation of total wine mannoprotein (but not grape-derived polysaccharide) with wine astringency as it relates to the progression of grape ripening [64]. However, in that instance, astringency was also correlated with wine tannin concentration, as also shown in our results. Based on our data, and the observation that the only substantive evidence that mannoprotein-tannin interactions may directly decrease astringency in wine have come from a multifactorial study, evidence for this effect is lacking and would need to be shown to exist following the (stable) addition of polysaccharide to red wine. Nevertheless, our data do support the idea that the relationship between polysaccharide, tannin and astringency may be non-linear, as proposed by [63] and this too warrants further investigation.

Interestingly, a negative association of both *opacity* and *astringency* with free mannose in wine was found. In terms of yeast strain effects, increased mannose may reflect differences in mannosidase activity induced by stress conditions, [65] or possibly variable uptake of mannose by lactic acid bacteria. In the case of astringency, elevated glucose was associated with lowered astringency in our PLS model. However, the concentration of both mannose and glucose in the wines was likely to be too low to confer to a significant sensory impact, and fructose, which was at higher concentrations in some wines (Table 4), was not significant in the PLS model.

## 4. Conclusions

In light of the fact that a diverse range of yeast options (including both *Saccharomyces* and non-*Saccharomyces* strains) is now available to the wine industry for red wine production, the presented study aimed to present a holistic picture of wine chemical and style outcomes for a single cultivar (Shiraz) and selection of commercial *S. cerevisiae* strains. It was evident from the wine sensory results that diverse styles were achievable from only 10 strains and that these were most strongly discriminated by the sensory attributes *opacity* and *astringency*. While many yeast-derived volatile compounds were strongly defined by the respective strains, the variance in their concentrations was not significant enough to account for sensory differences in aroma and flavour. Rather, key differences in wine aroma and flavour attributes introduced by yeast were driven by other factors not clearly defined by the current results. Indeed, aroma perception in red wines has been shown to involve interactive effects amongst several compound classes [66]. Importantly, yeast-derived changes in non-volatiles notably increased in polysaccharide (yeast and grape-derived) and tannin were useful in defining important changes in the sensory descriptor *astringency*. The colour attribute *opacity* could also be well-modelled by yeast-driven changes in tannin concentration and the analytical measure for wine colour density. Although important changes in red wine tannin and colour achieved by yeast strain selection have been previously described in the literature, this is the first time that the associated changes in textural and visual impacts have been clearly described.

Regarding the selection of yeast as a tool to modify wine style, it is important to note that a diverse range of wine styles are achievable, which are nonetheless acceptable to wine consumers, in particular for the Shiraz grape variety presented in this study [9]. Notably, it is cautionary that the attribute *astringency* may be less desirable, particularly at higher tannin concentrations and reported astringency perception levels than those reported in our results [9]. Depending on the grape source, and inherent phenolic extractability, yeast strains may be selected to moderate phenolic extraction rather than promote it. However, given the concomitant increases in *vegetal* aromas associated with some of the strains producing lowered astringency, selection of a strain which results in important increases in key positive sensory attributes would be advisable. Based on our results, a relevant observation is that strains which had higher *astringency* and *opacity* also tended to have concomitant increases in *dark fruit, floral* and *viscosity* which are attributes known to be relevant to quality in multiple wine styles [9]. In cases where *astringency* is deemed excessive, post-fermentative fining, as opposed to yeast strain selection, may produce more consistently beneficial outcomes.

## Figures and Tables

**Table 1:** Commercial *Saccharomyces cerevisiae* strains and their hybrids, used for Shiraz winemaking.

Strain	Code	Strain	Supplier
Rhone 2323	2323	*Saccharomyces cerevisiae* var *cerevisiae*	Lallemand
AWRI 1503	1503	*Saccharomyces cerevisiae* × *Saccharomyces kudriavzevii* hybrid	AB Mauri
AWRI 796	796	*Saccharomyces cerevisiae* var. *cerevisiae*	AB Mauri
Enoferm BDX	BDX	*Saccharomyces cerevisiae* var. *cerevisiae*	Lallemand
Lalvin CLOS	CLOS	*Saccharomyces cerevisiae* var. *cerevisiae*	Lallemand
Lalvin EC1118	EC1118	*Saccharomyces cerevisiae* var *bayanus*	Lallemand
Zymaflore F15	F15	*Saccharomyces cerevisiae* var. *cerevisiae*	Laffort
Anchor NT50	NT50	*Saccharomyces cerevisiae* hybrid	Oenobrands
Zymaflore RX60	RX60	*Saccharomyces cerevisiae* var *cerevisiae*	Laffort
Uvaferm HPS	HPS	*Saccharomyces cerevisiae* var *cerevisiae*	Lallemand

**Table 2:** Effect of yeast strain on basic wine compositional parameters (data show means compared by one-way ANOVA, where significant differences of *p* < 0.05 were compared by a post-hoc Student’s *t*-test, with significant differences within a row shown by different letters, ns = not significant).

Basic Wine Composition	Yeast Strain										ANOVA *p*-Value
	2323	1503	1796	BDX	CLOS	EC1118	F15	NT50	RX60	HPS	
Alcohol (%)	15.8 ^bc^	15.7 ^bcd^	15.2 ^e^	15.4 ^cde^	15.9 ^b^	15.9 ^b^	15.7 ^bcd^	15.3 ^de^	16.4 ^a^	15.2 ^e^	0.0006
Residual sugar (g/L)	0.45 ^b^	1.20 ^a^	0.48 ^b^	0.41 ^b^	0.51 ^b^	0.45 ^b^	0.51 ^b^	0.48 ^b^	0.38 ^b^	0.40 ^b^	<0.05
Glycerol (g/L)	9.9 ^f^	12.6 ^a^	11.0 ^d^	12.3 ^ab^	10.3 ^ef^	10.6 ^de^	11.5 ^c^	12.2 ^b^	10.0 ^f^	9.2 ^g^	<0.0001
Malic acid (g/L)	0.03	0.03	0.03	0.06	0.07	0.02	0.03	0.06	0.03	0.02	ns
Succinic acid (g/L)	1.01 ^e^	1.28 ^bc^	1.47 ^a^	1.47 ^a^	1.43 ^ab^	1.04 ^de^	1.32 ^abc^	1.19 ^cd^	1.07 ^de^	1.01 ^e^	<0.0001
Acetic acid (g/L)	0.24 ^bc^	0.20 ^cd^	0.30 ^a^	0.21 ^bcd^	0.24 ^bc^	0.24 ^b^	0.29 ^a^	0.19 ^d^	0.33 ^a^	0.31 ^a^	<0.0001
Titratable acidity (g/L)	5.97	6.00	5.87	6.37	6.07	6.03	5.80	6.27	5.87	6.03	ns
pH	3.57 ^abc^	3.48 ^cd^	3.65 ^ab^	3.54 ^bcd^	3.62 ^ab^	3.55 ^abcd^	3.64 ^ab^	3.46 ^d^	3.65 ^a^	3.45 ^d^	0.002

**Table 3:** Effect of yeast strain on wine tannin and colour composition (data show means compared by one-way ANOVA, where significant differences of *p* < 0.05 were compared by a post-hoc Student’s *t*-test, with significant differences within a row are shown by different letters, ns = not significant).

Compositional measure of wine tannin or colour	Yeast Strain										ANOVA *p*-Value
	2323	1503	1796	BDX	CLOS	EC1118	F15	NT50	RX60	HPS	
Tannin											
Tannin concentration (mg/L)	1508 ^a^	906 ^d^	1120 ^bcd^	976 ^cd^	1361 ^ab^	1238 ^ab^	1273 ^ab^	902 ^d^	1175 ^bc^	1129 ^bcd^	<0.01
Molecular mass (g/mol, subunit) ^1^	1936 ^a^	1603 ^c^	1895 ^a^	1788 ^ab^	1883 ^a^	1759 ^abc^	1890 ^a^	1798 ^ab^	1712 ^bc^	1883 ^a^	<0.05
Molecular mass (g/mol, 50% elution by GPC) ^2^	1693 ^ab^	1553 ^bcd^	1575 ^bcd^	1536 ^cd^	1673 ^ab^	1730 ^a^	1631 ^abcd^	1501 ^d^	1614 ^abcd^	1655 ^abc^	<0.05
mDP (no units) ^3^	6.51 ^a^	5.41 ^c^	6.38 ^a^	6.02 ^ab^	6.33 ^a^	5.92 ^abc^	6.36 ^a^	6.06 ^ab^	5.76 ^bc^	6.33 ^a^	<0.01
Epigallocatechin (%)	30.1 ^a^	24.0 ^c^	29.2 ^a^	28.9 ^a^	28.8 ^a^	28.0 ^ab^	27.2 ^abc^	29.6 ^a^	24.7 ^bc^	29.7 ^a^	<0.05
Galloylation (%)	1.61 ^abc^	1.53 ^bc^	1.53 ^bc^	1.43 ^cd^	1.67 ^ab^	1.55 ^bc^	1.72 ^ab^	1.23 ^d^	1.80 ^a^	1.44 ^cd^	<0.01
Tannin mass conversion (%) ^4^	26.8	30.2	28.6	26.7	26.0	24.8	32.1	31.8	26.3	27.1	ns
Colour ^5^											
Total anthocyanin (mg/L)	338 ^a^	253 ^d^	321 ^ab^	284 ^cd^	307 ^abc^	289 ^bcd^	317 ^abc^	289 ^bcd^	284 ^bcd^	288 ^bcd^	<0.05
Wine colour density (SO_2_ corrected, A.U.)	12.53 ^a^	9.91 ^d^	11.46 ^abc^	10.45 ^bcd^	11.4 ^abc^	11.44 ^abc^	11.80 ^ab^	11.20 ^ab^	10.94 ^bcd^	10.34 ^cd^	<0.05
Hue (no units)	0.71	0.72	0.72	0.72	0.71	0.73	0.72	0.72	0.72	0.72	ns
Non-bleachable pigment (A.U.)	3.66 a	2.87 ^cd^	3.12 ^bcd^	2.79 ^d^	3.15 ^bcd^	3.32 ^ab^	3.19 ^abc^	3.23 ^abc^	3.24 ^abc^	2.82 ^cd^	<0.05

^1^ Molecular mass determined by using subunit composition from phloroglucinolysis. ^2^ Molecular mass determined at 50% elution by gel permeation chromatography (GPC). ^3^ Mean degree of polymerization. ^4^ Mass conversion based on % recovery of proanthocyanidin by phloroglucinolysis as a proportion of tannin concentration by methyl cellulose precipitation. ^5^ Wine colour parameters determined according to the calculations outlined in Mercurio et al. [40].

**Table 4:** Effect of yeast strain on wine polysaccharide and monosaccharide composition (data show means compared by one-way ANOVA, where significant differences of *p* < 0.05 were compared by a post-hoc Student’s *t*-test, with significant differences within a row shown by different letters, ns = not significant).

Free and Soluble Polysaccharide-Associated Monosaccharide Composition	Yeast Strain										ANOVA *p*-Value
	2323	1503	1796	BDX	CLOS	EC1118	F15	NT50	RX60	HPS	
Polysaccharide											
Total polysaccharide (mg/L)	678 ^a^	486 ^cd^	576 ^b^	511 ^bcd^	430 ^cd^	474 ^cd^	427 ^d^	451 ^cd^	518 ^bc^	467 ^cd^	<0.01
Mannose (mg/L)	140 ^a^	109 ^bc^	131 ^a^	127 ^ab^	93 ^c^	99 ^c^	97 ^c^	102 ^c^	103 ^c^	106 ^c^	<0.01
Rhamnose (mg/L)	63.6 ^a^	48.4 ^c^	56.3 ^ab^	49.3 ^bc^	41.0 ^de^	44.8 ^cde^	38.8 ^e^	46.8 ^cd^	49.3 ^bc^	47.8 ^cd^	<0.001
Glucuronic acid (mg/L)	14.2 ^a^	12.1 ^abc^	13.5 ^a^	11.9 ^abc^	10.1 ^c^	10.9 ^bc^	10.5 ^bc^	13.8 ^a^	12.3 ^ab^	12.5 ^ab^	<0.05
Galacturonic acid (mg/L)	116 ^a^	58 ^bcd^	64 ^bcd^	56 ^cd^	51 ^cd^	69 ^bc^	46 ^d^	49 ^d^	75 ^b^	54 ^cd^	<0.001
Glucose (mg/L)	45.0 ^ab^	25.8 ^c^	45.7 ^a^	35.7 ^abc^	33.4 ^bc^	31.9 ^c^	32.0 ^c^	29.6 ^c^	31.3 ^c^	28.8 ^c^	<0.05
Galactose (mg/L)	129 ^a^	103 ^bc^	120 ^ab^	111 ^abc^	96 ^c^	100 ^c^	94 ^c^	99 ^c^	104 ^bc^	101 ^c^	<0.05
Xylose (mg/L)	4.36 ^a^	2.51 ^bcd^	3.03 ^bc^	2.86 ^bcd^	2.22 ^cd^	2.79 ^bcd^	3.33 ^b^	2.72 ^bcd^	3.20 ^b^	2.10 ^d^	<0.01
Arabinose (mg/L)	163 ^a^	124 ^bc^	140 ^b^	115 ^cd^	101 ^d^	112 ^cd^	102 ^d^	106 ^cd^	136 ^b^	112 ^cd^	<0.001
Fucose (mg/L)	2.75	2.73	3.56	2.47	2.67	2.64	2.95	3.02	3.22	2.78	ns
Free Monosaccharides^1^											
Mannose (mg/L)	13.8 ^cde^	20.4 ^a^	16.8 ^bc^	16.7 ^bc^	17.0 ^b^	16.4 ^bcd^	13.6 ^de^	16.5 ^bc^	12.4 ^e^	17.4 ^b^	<0.001
Ribose (mg/L)	19.6 ^abc^	21.1 ^ab^	24.1 ^a^	21.2 ^ab^	12.8 ^d^	14.5 ^cd^	18.5 ^bc^	23.9 ^a^	17.9 ^bc^	12.2 ^d^	<0.001
Rhamnose (mg/L)	20.9 ^abcd^	17.2 ^bcde^	22.1 ^ab^	26.5 ^a^	14.1 ^e^	14.7 ^de^	17.1 ^bcde^	21.0 ^abc^	15.4 ^cde^	17.1 ^bcde^	<0.01
Galacturonic acid (mg/L)	1161 ^a^	1055 ^b^	879 ^c^	633 ^d^	511 ^e^	874 ^c^	543 ^e^	842 ^c^	1135 ^ab^	875 ^c^	<0.0001
Galactose (mg/L)	89.0	88.9	92.3	83.9	82.1	83.7	85.8	87.3	85.6	84.2	ns
Xylose (mg/L)	6.4 ^bcd^	7.3 ^abc^	6.3 ^cd^	5.6 ^d^	6.7 ^bcd^	8.1 ^ab^	8.6 ^a^	6.3 ^cd^	8.8 ^a^	5.3 ^d^	<0.01
Arabinose (mg/L)	19.1 ^bc^	17.8 ^bc^	21.5 ^ab^	22.1 ^ab^	16.4 ^bcd^	17.1 ^bc^	12.7 ^cd^	27.0 ^a^	10.8 ^d^	17.6 ^bc^	<0.001
Fucose (mg/L)	8.72 ^ab^	9.67 ^a^	8.34 ^ab^	8.63 ^ab^	7.52 ^bc^	8.12 ^b^	7.27 ^bc^	8.29 ^ab^	6.41 ^c^	7.99 ^b^	<0.01
Glucose (mg/L)	39.0 ^f^	76.3 ^cd^	99.2 ^ab^	88.7 ^bc^	48.2 ^ef^	53.9 ^de^	58.9 ^a^	109.1 ^a^	0.0 ^g^	60.6 ^de^	<0.0001
Fructose (mg/L)	372 ^b^	1118 ^a^	382 ^b^	325 ^b^	459 ^b^	452 ^b^	447 ^b^	366 ^b^	376 ^b^	343 ^b^	<0.05

^1^ excluding glucuronic acid.

**Table 5:** Effect of yeast strain on the polysaccharide composition (as mg/g dry weight of total and individual monosaccharides) of marcs and lees post-fermentation (data show means compared by one-way ANOVA, where significant differences of *p* < 0.05 were compared by a post-hoc Student’s *t*-test, with significant differences within a row shown by different letters, ns = not significant).

Monosaccharide Composition of Insoluble Polysaccharides	Yeast Strain								ANOVA *p*-Value
	2323	1503	1796	BDX	CLOS	EC1118	F15	RX60	
Marc skin cell wall composition ^1^									
Total polysaccharide	286 ^bcd^	266 ^def^	309 a^b^	277 ^cde^	260 ^ef^	249 ^f^	314 ^a^	302 ^abc^	<0.001
Mannose	27.2 ^b^	25.9 ^b^	37.2 ^a^	29.3 ^b^	28.5 ^b^	28.7 ^b^	29.8 ^b^	26.5 ^b^	<0.05
Rhamnose	7.39	7.72	9.11	8.44	7.73	7.41	7.85	6.50	ns
Glucuronic acid	9.07 ^a^	5.75 ^bc^	8.51 ^a^	8.60 ^a^	4.50 ^c^	6.94 ^abc^	7.39 ^ab^	5.66 ^bc^	<0.05
Galacturonic acid	38.2 ^c^	40.9 ^bc^	40.2 ^bc^	37.2 ^c^	35.6 ^c^	38.0 ^c^	50.3 ^a^	45.2 ^ab^	<0.01
Glucose	140 ^ab^	128 ^b^	156 ^a^	138 ^ab^	124 ^bc^	104 ^c^	154 ^a^	152 ^a^	<0.001
Galactose	20.4	18.3	18.5	17.7	18.5	19.4	20.0	20.8	ns
Xylose	16.4	15.7	14.7	15.3	15.4	16.8	17.5	17.7	ns
Arabinose	27.5 ^ab^	23.8 ^c^	24.7 ^bc^	23.1 ^c^	25.7 ^abc^	27.6 ^a^	27.0 ^ab^	26.8 ^ab^	<0.05
Lees composition									
Total polysaccharide	205 ^a^	142 ^b^	210 ^a^	197 ^a^	194 ^a^	161 ^b^	192 ^a^	162 ^b^	<0.0001
Mannose	77 ^bcd^	55 ^e^	93 ^a^	86 ^ab^	80 ^abc^	68 ^cde^	80 ^abcd^	65 ^de^	<0.01
Rhamnose	2.58	2.31	2.51	2.44	2.20	2.47	2.06	2.19	ns
Glucuronic acid	1.87 ^abc^	1.99 ^ab^	1.69 ^bcd^	1.89 ^abc^	2.18 ^a^	1.57 ^cd^	1.52 ^cd^	1.32 ^d^	<0.01
Galacturonic acid	3.42 ^ab^	4.18 ^a^	3.46 ^ab^	3.53 ^ab^	3.14 ^bc^	2.53 ^c^	2.63 ^c^	2.57 ^c^	<0.01
Glucose	104 ^a^	60 d	93 ^ab^	88 ^b^	92 ^b^	72 ^cd^	93 ^ab^	75 ^c^	<0.0001
Galactose	6.98	8.01	6.85	6.33	6.34	5.97	6.28	6.85	ns
Xylose	1.72 ^ab^	1.83 ^a^	1.34 ^b^	1.44 ^b^	1.42 ^b^	1.59 ^ab^	0.71 ^c^	1.50 ^ab^	<0.001
Arabinose	7.12 ^ab^	7.64 ^a^	7.19 ^ab^	6.66 ^bc^	6.28 ^bc^	6.10 ^c^	6.17 ^c^	6.73 ^abc^	<0.05
Fucose	0.87 ^abc^	0.87 ^abc^	0.71 ^bcd^	0.91 ^ab^	0.91 ^ab^	1.03 ^a^	0.69 ^cd^	0.57^d^	<0.01

^1^ fucose not detected.

**Table 6:** Partial least squares (PLS1) regression analysis of the wine sensory attributes opacity and astringency (Y) showing the total principal component (PC) number in the model, *R*-squared values for calibration (cal) and validation (val), root mean squared error of prediction (RMSE) and the explained variance (%) for all compositional X variables, and sub-sets of significant X variables.

Variables Included in PLS1 Model	Opacity PLS1 Model							Astringency PLS1 Model						
	PC No	*R* ^2^_cal_	*R* ^2^_val_	RSME_cal_	RSME_val_	X (%)	Y (%)	PC No	*R* ^2^_cal_	*R* ^2^_val_	RSME_cal_	RSME_val_	X (%)	Y (%)
All variables	2	0.93	0.70	0.17	0.39	31	93	2	0.88	0.60	0.08	0.15	32	87
Significant variables *	1	0.91	0.89	0.20	0.23	66	91	1	0.82	0.76	0.10	0.12	37	82
Significant variables * excluding pH and TA	1	0.90	0.88	0.21	0.24	72	90	1	0.81	0.75	0.10	0.12	38	81

* identified using uncertainty test.

## Supplementary Materials

The following are available online at https://www.mdpi.com/2218-273X/9/9/466/s1, Table S1. Attributes, definitions and reference standards evaluated by sensory panelists; Figure S1 Must sugar and during the initial phase of fermentation; Table S2. Fermentation products in Shiraz wines prepared using 10 commercial yeast strains; Table S3. Low molecular weight volatile sulfur compounds in Shiraz wines; Figure S2. Principal component analysis of wine volatile composition; Figure S3. Bound tannin concentration in lees and marcs; Figure S4. Size exclusion profiles of wine polysaccharides; Figure S5. Principal components analysis of the carbohydrate composition of monosaccharides, polysaccharides, lees and marcs. Table S4. Tannin, colour and polysaccharides in model fermentations Table S5. Sensory attributes of Shiraz wines made with different yeast strains.
