# Supplementary material for: Commercial Saccharomyces cerevisiae Yeast Strains Significantly Impact Shiraz Tannin and Polysaccharide Composition with Implications for Wine Colour and Astringency
Source: Biomolecules. 2019 Sep 9;9(9):466. doi: 10.3390/biom9090466 (PMC6770880; doi:10.3390/biom9090466)
Supplement: Supplementary file 1 [file biomolecules-09-00466-s001.pdf]

Table S1. Attributes, definitions and reference standards evaluated by panellists in formal sessions for the wines.

Attribute	Definition/Synonyms	Standard composition
Appearance		
Opacity	The degree to which light is not allowed to pass through a sample	
Aroma		
Overall fruit	Intensity of the fruit aromas in the sample	
Red fruits	Intensity of the aroma of red fruits and berries: raspberries, strawberries, cranberries, redcurrants	3 x frozen raspberries, Sara Lee brand
Dark fruits	Intensity of the aroma of dark fruits and berries: blackberries, plums, black currants, cherries	3 x frozen blueberries, 1 x frozen blackberry, Sara Lee brand
Confection	Intensity of the aroma of confection: raspberry lollies, musk lollies	3 x raspberry lollies, Natural Confectionary Company brand
Floral	Intensity of the aroma of flowers: violets, roses	40 µL of 100 mg/L Linalool, 25 µL of 200 mg/L 2-phenyl ethanol
Green	Intensity of the aroma of green stalks, leaves, grass, green beans, green capsicum, geranium	1 tomato stalk and 1 cm cut up fresh green bean, no wine
Herbal	Intensity of the aroma of herbs: mixed herbs, mint, menthol	1 mint leaf, crushed and 1/8 tsp dried mixed herbs, no wine
Vegetal	Intensity of the aroma of various vegetables, cooked vegetables	1 tbsp vegetable water, tin of cooked green beans, Edgell brand
Vanilla/Chocolate	Intensity of the aroma of vanilla and chocolate.	1/8 tsp vanilla paste, Queen brand
Earthy	Intensity of the aroma of wet earth, organic matter, compost, mushrooms, beetroot.	25 µL of 4 mg/L geosmin
Pungent	Intensity of the aroma and effect of alcohol	4 ml ethanol
Palate		
Overall fruit	Intensity of fruit flavours in the sample	
Red fruits	Intensity of the flavour of red fruits and berries: raspberries, strawberries, cranberries	
Dark Fruits	Intensity of the flavour of various dark fruits: blackberries, currants, plums, cherries	
Green	Intensity of the flavour of green stalks, green capsicum, green bean, herbs.	
Vanilla	Intensity of the flavour of vanilla, chocolate	
Viscosity	Perception of the body, weight or thickness of the wine in the mouth. Low, watery; high, oily, thick.	
Sweet	Intensity of the taste of sucrose	
Salt	Intensity of the taste of salt	
Acid	Intensity of acid taste in the mouth, including aftertaste	
Hotness	The intensity of alcohol hotness (burning) perceived in-mouth or after expectoration.<br>Low, warm; high, hot, burning.	
Astringency	Drying and mouth-puckering sensation.<br>Low, coating teeth; medium, mouth coating/drying; high, puckering, lasting astringency.	
Bitter	The intensity of bitter taste perceived in the mouth, or after expectoration.	
Fruit AT	The lingering fruit flavour perceived in the mouth after expectorating.	

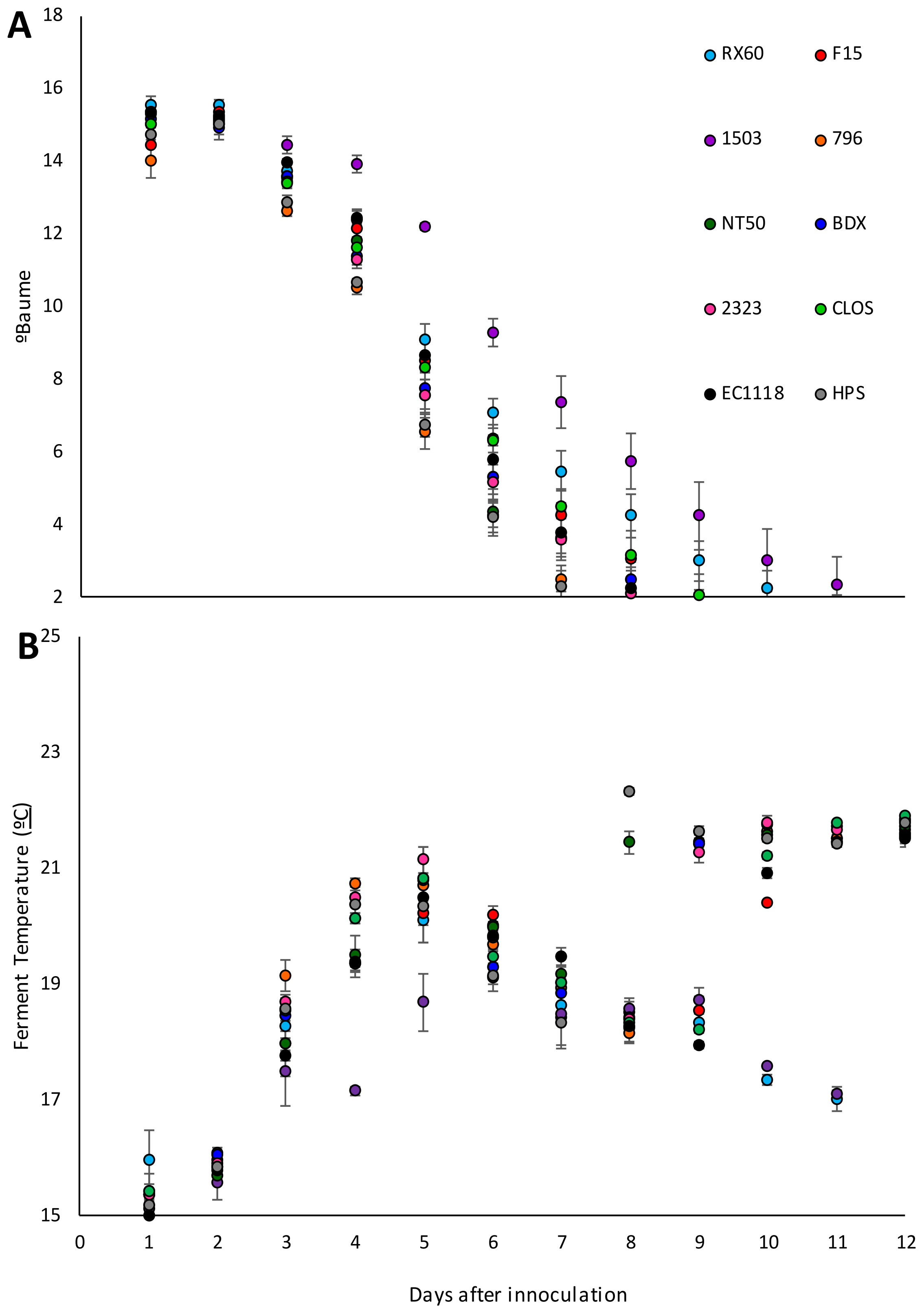

Figure S1. Changes in (**A**) must sugar and (**B**) ferment temperature during the initial phase of fermentation of 10 commercial yeast strains (pressing occurred at 2 °Baume).

Table S2. Fermentation products in Shiraz wines prepared using 10 commercial yeast strains analysed at 2 years of wine age (data show means compared by one-way ANOVA, where significant differences of $p < 0.01$ were compared by a post-hoc Student's *t*-test, significant differences within a row are shown by different letters).

	unit	Yeast strain										ANOVA *P* value
		2323	1503	1796	BDX	CLOS	EC1118	F15	NT50	RX60	HPS	
Acetate esters												
ethyl acetate	mg/L	51cd	41f	60ab	43ef	48de	47de	55bc	40f	63a	63a	<0.0001
hexyl acetate	µg/L	17.2cde	11.6e	37.2a	12.6e	14.6de	13.0de	22.9bc	15.5de	18.4cd	25.5b	<0.0001
2-methylpropyl acetate	µg/L	609c	1255a	677c	707c	719c	968b	730c	1002b	819bc	592c	<0.001
2-phenyl ethyl acetate	µg/L	61b	85a	78a	90a	46cd	63b	53bc	59bc	38d	58bc	<0.0001
3-methylbutyl acetate	µg/L	941b	382d	1292a	913b	665c	525cd	912b	523cd	603c	1057b	<0.0001
2-methylbutyl acetate	µg/L	269a	134c	262a	280a	184b	182b	220b	139c	184b	298a	<0.0001
Ethyl esters												
ethyl butanoate	µg/L	436ab	365bc	456a	328c	307c	292c	348bc	337c	455a	420ab	<0.01
ethyl propanoate	µg/L	242d	361a	330b	258cd	257cd	317b	236d	324b	274c	241d	<0.0001
ethyl hexanoate	µg/L	58bc	58b	70a	42d	55bc	48cd	58b	58bc	76a	53bc	<0.0001
ethyl octanoate	µg/L	53bc	55b	69a	37d	50bc	44cd	58b	56b	71a	53b	<0.0001
ethyl decanoate	µg/L	7.4de	10.0bc	10.5bc	6.4e	7.1e	6.8e	11.0ab	9.2cd	12.2a	9.4c	<0.0001
ethyl 2-methylbutanoate	µg/L	47ab	49a	32c	40b	30c	44ab	31c	47ab	26c	40ab	<0.0001
ethyl-3-methylbutanoate	µg/L	82a	71ab	47de	80a	43e	70ab	55cd	67bc	39e	69ab	<0.0001
ethyl 2-methylpropanoate	µg/L	211a	108d	110d	214a	115d	104d	124d	152bc	130cd	174b	<0.0001
Higher alcohols												<0.0001
butanol	mg/L	1.0bcd	1.2abc	1.1abc	0.7d	0.9cd	1.4a	0.8d	1.2ab	1.0bcd	1.3ab	<0.01
2-methylbutanol	mg/L	109b	97c	78f	158a	86de	94cd	94cd	80ef	73f	92cd	<0.0001
3-methylbutanol	mg/L	267b	234cd	213ef	385a	220de	204f	238c	188gh	183h	202fg	<0.0001
hexanol	mg/L	4.8bcd	6.0a	5.2b	4.9bcd	4.7cd	5.0bcd	5.1bc	4.1e	6.4a	4.5de	<0.0001
2-phenyl ethyl ethanol	mg/L	48.1cde	90.3a	43.3ef	88.3a	48.9cd	68.7b	45.0de	52.0c	39.3fg	35.7g	<0.0001
2-methylpropanol	mg/L	69b	48d	41e	123a	52cd	40e	56c	42e	55c	56c	<0.0001
Volatile acids												
acetic acid	mg/L	244bc	198cd	295a	208bcd	237bc	244b	293a	193d	330a	310a	<0.0001
butanoic acid	mg/L	12.2	12.0	12.1	11.6	11.9	11.7	11.8	11.9	11.6	11.8	ns
propanoic acid	mg/L	1.7de	2.1abcd	2.2ab	1.9cde	1.9bcde	2.3a	1.8de	2.2abc	1.7e	1.8cde	<0.01
hexanoic acid	mg/L	1.9bcd	2.1b	2.7a	1.4e	1.9bcde	1.6de	2.2b	2.1bc	2.7a	1.7cde	<0.0001
octanoic acid	mg/L	1.9cd	1.9c	2.5a	1.4e	1.8cd	1.6de	2.0bc	2.0bc	2.2b	1.9c	<0.0001
decanoic acid	µg/L	417bcd	419bc	639a	297cde	276de	233e	393bcd	439b	573a	392bcd	<0.0001
2-methyl butanoic acid	µg/L	890a	831ab	632e	770bc	654de	788b	761bc	781bc	641e	713cd	<0.0001
3-methyl butanoic acid	µg/L	1389a	1080b	906cd	1302a	777de	1103b	1100b	997bc	764e	1031bc	<0.0001
2-methyl propanoic acid	µg/L	1012a	416d	717bc	1015a	676bc	541cd	807ab	634bcd	722bc	967a	<0.001

ns, not significant

Table S3. Low molecular weight volatile sulfur compounds in Shiraz wines prepared using 10 commercial yeast strains analysed at 2 years of wine age (data show means compared by one-way ANOVA, where significant differences of $p < 0.05$ were compared by a post-hoc Student's *t*-test, significant differences within a row are shown by different letters).

	Yeast strain										ANOVA *P* value
	2323	1503	796	BDX	CLOS	EC1118	F15	NT50	RX60	HPS	
Hydrogen sulfide (μg/L)	2.53de	3.35bcd	3.02cde	3.73abc	4.15ab	4.27a	3.37bcd	3.80abc	2.28e	3.23cd	<0.01
Methanethiol (μg/L)	2.63d	3.69a	3.09bcd	3.83a	3.19bc	3.14bcd	3.45ab	3.52ab	2.84cd	2.66d	<0.001
Dimethyl sulfide (μg/L)	53.1b	48.8b	48.6b	136.7a	49.5b	45.9b	42.5b	47.2b	46.7b	50.5b	<0.0001
Carbon disulfide (μ/L)	4.11ab	9.63ab	1.83b	2.58b	5.80ab	7.11ab	9.76a	2.85b	9.26a	2.01b	<0.05
Diethyl sulfide (μ/L)	0.22bc	0.30ab	0.25abc	0.23bc	0.21c	0.31ab	0.25abc	0.21c	0.32a	0.32a	<0.05
Methylthioacetate (μ/L)	18.7a	12.0bc	9.6cd	18.9a	14.4b	7.4d	11.9bc	9.6cd	8.2d	12.3bc	<0.0001

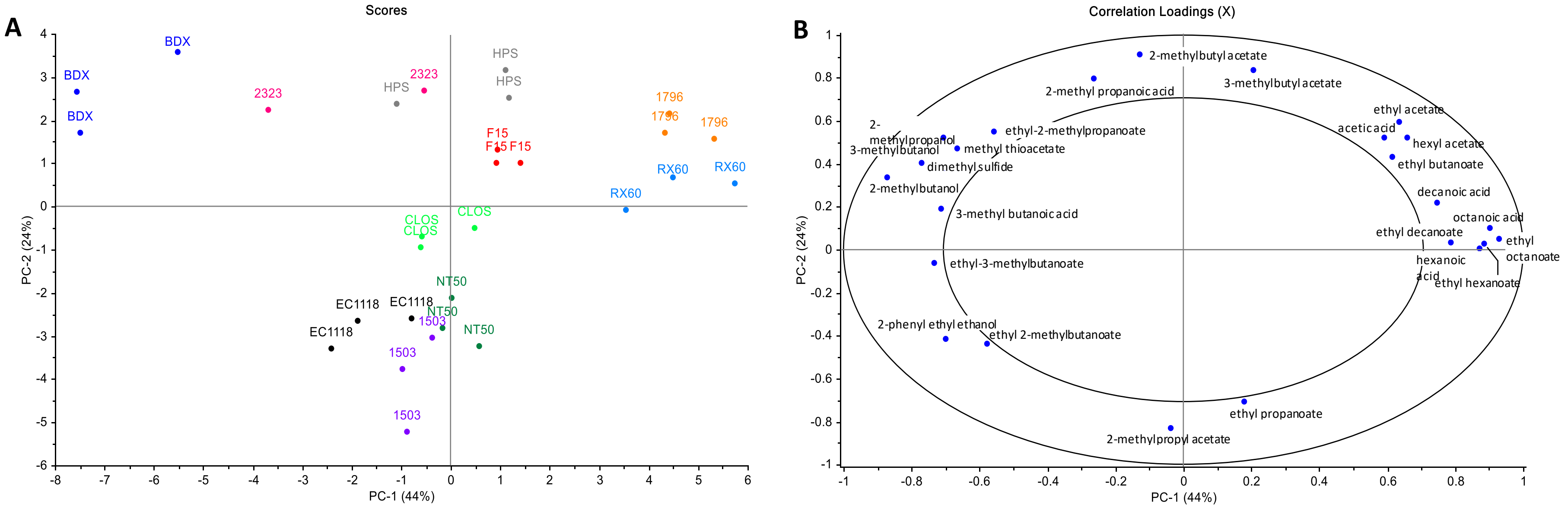

Figure S2. Principal component analysis of the composition of wine fermentation products and low molecular weight volatile sulfur compounds at 2 years of wine age (**A**) Scores plot showing each yeast strain as a different colour; (**B**) Correlation loadings plot for volatile compounds (triplicate wines shown for all strains except 2323 for which duplicates are shown).

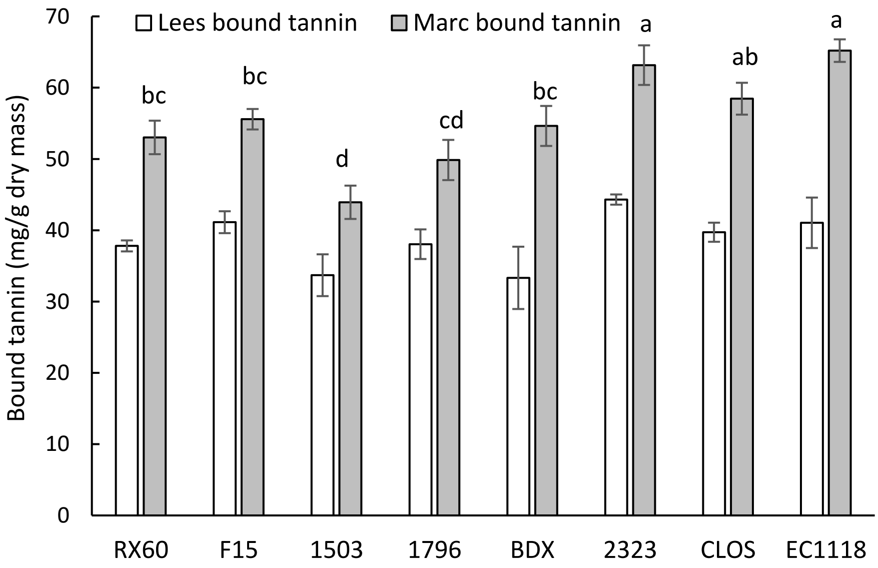

Figure S3. Bound tannin concentration recovered in the lees and marcs of a sub-set of 8 yeast strains determined by direct phloroglucinolysis (data show means compared by one-way ANOVA, where significant differences of $p < 0.01$ were compared by a post-hoc Student's *t*-test, significant differences within a row are shown by different letters).

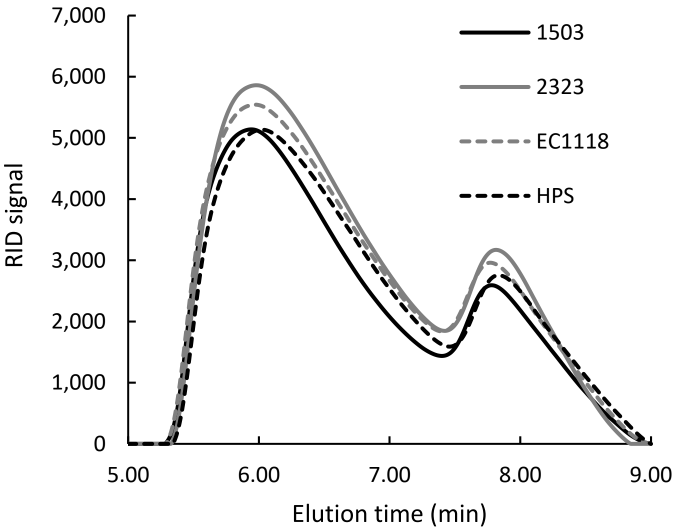

Figure S4. Size exclusion profiles of polysaccharides isolated from Shiraz wines prepared from four yeast strains.

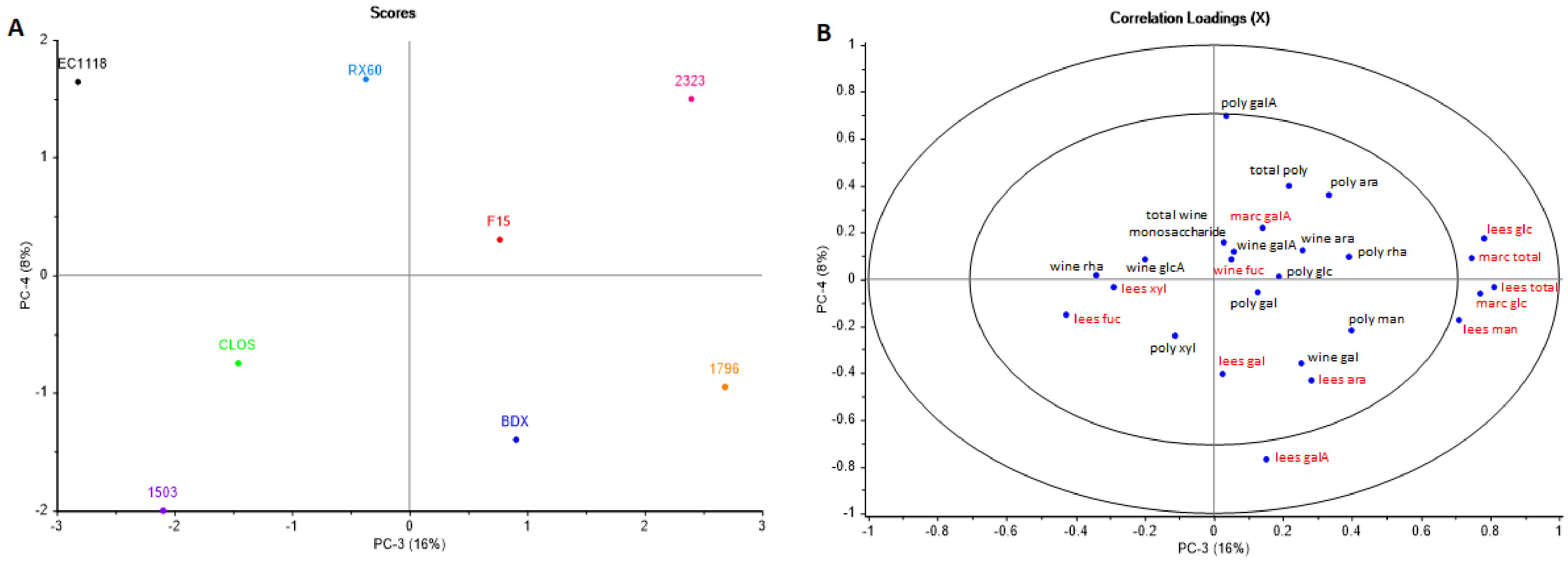

Figure S5. Principal component analysis showing PCs 3 and 4 of the composition of wine soluble (wine) polysaccharide-associated (poly) monosaccharides, and hydrolytically-released monosaccharides from purified lees and marcs of 8 commercial yeast strains (**A**) Scores plot showing each yeast strain as a different colour; (**B**) Correlation loadings plot for wine composition (black) and lees or marc composition (red). Abbreviations: man = mannose, rha = rhamnose, glcA = glucuronic acid, galA = galacturonic acid, glc = glucose, gal = galactose, xyl = xylose, ara = arabinose, fuc = fucose.

Table S4. Concentration of tannin, colour and wine-soluble and lees-associated polysaccharide recovered from model fermentations conducted by 5 yeast strains in Shiraz juice with anthocyanin and tannin added to 550 mg/L and 1.2 g/L respectively (data show means compared by one-way ANOVA, where significant differences of $p < 0.05$ were compared by a post-hoc Student's *t*-test, significant differences within a row are shown by different letters).

	Yeast strain					ANOVA P value
	2323	1503	BDX	EC1118	HPS	
Tannin (mg/L)	960	911	879	837	915	ns
Total anthocyanin (mg/L)	378	370	367	382	330	ns
Wine colour density (A.U.)	6.7	7.2	7.1	7.7	7.0	ns
Non-bleachable pigment (A.U.)	1.36	1.43	1.37	1.39	1.53	ns
Total wine polysaccharide (mg/L)	434	431	450	467	505	ns
Mannose (mg/L)	73c	75bc	73c	97a	92ab	<0.05
Rhamnose (mg/L)	25.9ab	20.4c	24.8b	27.2a	25.7ab	<0.01
Glucuronic acid (mg/L)	9.3a	6.4c	8.2ab	7.4bc	7.0bc	<0.05
Galacturonic acid (mg/L)	30.6a	25.7c	26.9bc	27.5b	26.6bc	<0.001
Glucose (mg/L)	105	124	131	117	177	ns
Galactose (mg/L)	84	79	82	82	77	ns
Xylose (mg/L)	8.0ab	7.7bc	8.4a	7.9ab	7.4c	<0.05
Arabinose (mg/L)	95	89	92	96	88	ns
Fucose (mg/L)	3.5bc	2.5d	3.3c	4.2a	4.0ab	<0.0001
Total lees (g D. wt/90 mL)	0.36a	0.26c	0.28b	0.28c	0.32b	<0.0001
Total lees polysaccharide (mg/L)*	424a	315c	347bc	309c	390b	<0.01
Mannose (mg/L)	131	146	129	146	149	ns
Rhamnose (mg/L)	1.4	0.9	0.5	0.7	1.2	ns
Glucuronic acid (mg/L)	1.8	1.2	1.1	1.5	1.3	ns
Galacturonic acid (mg/L)	2.5	2.4	1.7	1.7	1.7	ns
Glucose (mg/L)	281a	158c	211bc	153c	230b	<0.001
Galactose (mg/L)	2.6	2.8	1.6	2.5	3.2	ns
Arabinose (mg/L)	2.3	2.6	1.8	2.5	2.9	ns
Fucose (mg/L)	1.2	1.0	0.5	0.7	0.9	ns

ns, not significant; *xylose not detected

Table S5. Mean values and LSD values from sensory analysis of the Shiraz wines made with different yeast strains.

Sensory attribute	Yeast strain										ANOVA *P* value	LSD
	2323	1503	1796	BDX	CLOS	EC1118	F15	NT50	RX60	HPS		
Appearance												
Opacity	5.62	3.52	4.99	4.18	5.15	4.75	4.86	4.15	4.72	4.05	<0.0001	0.29
Aroma												
Overall fruit aroma	3.66	3.34	3.80	3.44	3.59	3.73	3.54	3.51	3.63	3.66	ns	ns
Red fruit aroma	3.02	2.93	3.13	2.96	2.83	3.12	3.10	2.91	3.11	3.08	ns	ns
Dark fruit aroma	2.96	2.54	3.12	2.82	2.94	3.04	2.65	2.68	2.78	2.53	<0.001	0.33
Confection aroma	1.38	1.19	1.70	1.26	1.30	1.57	1.62	1.43	1.50	1.57	ns	ns
Floral aroma	1.58	1.29	1.64	1.40	1.46	1.56	1.64	1.43	1.46	1.08	ns*	ns
Green aroma	1.53	1.46	1.51	1.51	1.64	1.48	1.50	1.40	1.48	1.42	ns	ns
Herbal aroma	1.89	1.63	1.54	1.96	1.79	1.75	1.73	1.42	1.80	1.56	<0.01	0.30
Vegetal aroma	1.40	1.92	1.46	2.43	1.61	1.87	1.57	2.03	1.49	1.85	<0.02	0.53
Vanilla/Chocolate aroma	1.80	1.50	1.80	1.33	1.55	1.69	1.43	1.48	1.62	1.52	ns*	ns
Earthy aroma	2.56	2.52	2.25	2.42	2.41	2.29	2.32	2.40	2.32	2.29	ns	ns
Pungent aroma	2.62	2.57	2.74	2.84	2.74	2.65	2.45	2.46	2.53	2.61	ns	ns
Palate												
Overall fruit flavour	3.95	3.86	4.12	3.80	3.92	4.01	4.02	3.90	4.02	3.92	ns	ns
Red fruit flavour	3.05	3.26	3.40	3.09	3.09	3.21	3.26	3.23	3.18	3.21	ns	ns
Dark fruit flavour	3.36	3.08	3.33	3.09	3.38	3.37	3.29	3.28	3.42	3.12	ns	ns
Green flavour	1.78	1.75	2.00	2.10	2.01	1.85	1.98	1.79	2.07	1.88	ns	ns
Vanilla flavour	1.74	1.77	1.81	1.63	1.71	1.72	1.75	1.70	1.82	1.75	ns	ns
Viscosity	2.94	2.73	2.77	2.80	2.79	2.71	2.89	2.72	2.85	2.72	<0.05	0.15
Sweet	0.83	1.30	0.98	0.80	0.74	1.01	0.91	1.02	0.81	0.93	<0.05	0.30
Salt	1.66	1.37	1.60	1.83	1.61	1.14	1.46	1.45	1.67	1.52	<0.05	0.36
Acid	3.31	3.12	3.21	3.36	3.34	3.14	3.36	3.31	3.25	3.36	ns	ns
Astringency	4.38	3.67	4.08	4.07	4.09	4.16	4.09	3.69	4.13	3.97	<0.01	0.34
Hotness	3.32	3.15	2.88	3.28	2.99	3.03	3.51	3.04	3.29	3.13	<0.01	0.32
Bitter	1.87	1.53	1.75	1.94	1.58	1.98	1.94	1.68	1.70	1.74	<0.01	0.26
Fruit aftertaste	3.32	3.51	3.39	3.19	3.38	3.52	3.34	3.31	3.34	3.28	ns	ns

ns, not significant to the 5% confidence interval; ns*, significant to the 10% confidence interval, $P<0.1$; LSD, Fisher's Least Significant Difference
